# *Schistosoma* Transmission in a Dynamic Seasonal Environment and its Impact on the Effectiveness of Disease Control

**DOI:** 10.1093/infdis/jiaa746

**Published:** 2020-12-02

**Authors:** Qimin Huang, David Gurarie, Martial Ndeffo-Mbah, Emily Li, Charles H King

**Affiliations:** 1 Department of Mathematics, Applied Mathematics and Statistics, Case Western Reserve University, Cleveland, Ohio, USA; 2 Center for Global Health and Diseases, School of Medicine, Case Western Reserve University, Cleveland, Ohio, USA; 3 Department of Veterinary and Integrative Biosciences, College of Veterinary and Biomedical Sciences, Texas A&M University, College Station, Texas, USA; 4 School of Public Health, Texas A&M University, College Station, Texas, USA; 5 Ascension St Vincent Indianapolis, Family Medicine Residency, Indianapolis, Indiana, USA; 6 Schistosomiasis Consortium for Operational Research and Evaluation, University of Georgia, Athens, Georgia, USA; 7 World Health Organization Collaborating Centre for Research and Training for Schistosomiasis Elimination, Cleveland, Ohio, USA

**Keywords:** mathematical modeling, *Schistosoma*, seasonal transmission, epidemiology, disease control

## Abstract

**Background:**

A seasonal transmission environment including seasonal variation of snail population density and human-snail contact patterns can affect the dynamics of *Schistosoma* infection and the success of control interventions. In projecting control outcomes, conventional modeling approaches have often ignored seasonality by using simplified intermediate-host modeling, or by restricting seasonal effects through use of yearly averaging.

**Methods:**

We used mathematical analysis and numerical simulation to estimate the impact of seasonality on disease dynamics and control outcomes, and to evaluate whether seasonal averaging or intermediate-host reduction can provide reliable predictions of control outcomes. We also examined whether seasonality could be used as leverage in creation of effective control strategies.

**Results:**

We found models that used seasonal averaging could grossly overestimate infection burden and underestimate control outcomes in highly seasonal environments. We showed that proper intraseasonal timing of control measures could make marked improvement on the long-term burden reduction for *Schistosoma* transmission control, and we identified the optimal timing for each intervention. Seasonal snail control, implemented alone, was less effective than mass drug administration, but could provide additive impact in reaching control and elimination targets.

**Conclusions:**

Seasonal variation makes *Schistosoma* transmission less sustainable and easier to control than predicted by earlier modeling studies.

## Teaser

We used mathematical models to evaluate the impact of seasonality on *Schistosoma* transmission. We found the seasonal variation makes *Schistosoma* transmission less sustainable, and intraseasonal timing of interventions could improve the long-term outcomes of control measures in such communities.

Schistosomiasis, caused by *Schistosoma* species parasites, is highly prevalent in the tropics. Like many infectious diseases, *Schistosoma* transmission is highly seasonal with a time-dependent transmission rate that varies during a year. Because schistosome parasites split their life cycle between humans (who harbor the adult forms of the parasite) and intermediate-host freshwater snails (which harbor and amplify the infectious larval forms), human water contact, area contamination with sewage, and the presence of competent snail species all play significant roles in defining local transmission [1]. The observed seasonal nature of *Schistosoma* transmission is often related to the variable abundance of intermediate host snail populations, which is linked to weather patterns (rainfall, temperature), and/or the resulting availability and frequency of human-snail contacts [2–5]. The mathematical model of *Schistosoma* transmission was first proposed by G. Macdonald [6] and later developed in different directions [7–10]. However, most mathematical models of *Schistosoma* transmission do not account for the effects of seasonality and this could significantly affect the accuracy of their predictions. A deeper understanding of the effects of transmission seasonality should provide additional insight into the optimal timing of interventions used in public health practice for schistosomiasis control. The current options for control include periodic mass administration of the drug praziquantel and, in some locations, control of intermediate host snails with the use of chemical molluscicides or environmental modification [11–14].

In mathematical models of *Schistosoma* transmission, one way to deal with seasonal variability has been to use seasonal averaging of environmental and behavioral inputs, making the resulting stationary Macdonald system amenable to direct analysis [15]. However, little effort has been put into assessing the effect of such seasonal averaging on the resulting predictions of disease-risk or control outcomes (some recent works have made such attempts, eg, [16]). In general, the use of averaging methods in nonstationary dynamical systems can be justified when such variability is marginal relative to the baseline mean state, or when the quantities of interest (eg, dynamic variables or outputs) depend linearly on variable inputs. Under these conditions, the averaged stationary model, with properly adjusted coefficients, is able to reproduce the approximate “mean” behavior of the nonstationary (time-periodic) system. However, for nonlinear disease transmission models in variable seasonal environments, the output histories can depart significantly from the expected “mean” behavior [16], because of highly influential seasonal effects on local transmission.

Another commonly used procedure in modeling vector-host transmission is a reduction of the coupled human-snail transmission cycle to a single host equation via quasiequilibration of the snail equation. This can be justified by the relatively short duration of snail lifespan, relative to human and worm, but requires more careful analysis, both for the basic Macdonald system and for its extensions.

In the present study, we aimed to perform a systematic assessment of seasonal variability and its implications for *Schistosoma* control predictions, including those for programs relying on mass drug administration (MDA) and/or molluscicide-based snail control to achieve their public health objectives.

## METHODS

We modeled seasonal variability as seasonal changes of snail abundance, knowing that multiple environmental factors (precipitation, temperature, etc. [3–5, 17–21]) can affect snail population density. In our modelling analysis, we used 2 types of snail population dynamics: (1) a prescribed periodic snail density function and (2) a dynamic snail population model based on the underlying periodic carrying capacity function, combined with estimates of snail reproduction rate and mortality. The key dimensionless input parameters of the model were the basic reproduction number ($R_{0}$), and the amplitude of seasonal variability. We used type 1 snail population models to explore the parameter space of the system and identify regions of sustained infection versus elimination. For control analysis, however, we employed the dynamic snail model (type 2) to account for abrupt changes in population due to molluscicide. We then explored different periodicity patterns to approximate seasonal snail population dynamics, as informed by empirical data from past field studies on snail abundance [4, 5, 17, 18, 22, 23]. Details of the models are provided in Supplementary Material.

### Modeling of a Stationary Transmission Environment and Derivation of $R_{0}$

The basic Macdonald model for *Schistosoma* transmission combines 2 variables: mean worm burden (MWB) w(t) of human hosts, and infected snail prevalencey(t), coupled by a system of differential equations:


$$\begin{array}{l} \begin{array}{ll} & \frac{dw}{dt}=A\;y-\gamma \;w\\ & \frac{dy}{dt}=B\;w\left(1-y\right)-\nu y \end{array} \end{array}$$ (1)

Here, transmission coefficient *A* represents mean rate of worm accumulation per human host and *B* the force of snail infection [15, 24, 25]. CoefficientA is proportional to the snail density coefficient, *N*, coefficient Bdepends on the human population size *H*, and its infectivity (egg shedding by mated worm pairs in the human population). Both coefficients are proportional to human-sail contact rates. Coefficients γ and ν are the natural mortality rates for adult worms and snails. We took γ=0.25/year and allowed ν to vary from 6 to 2 per year (equivalent to a 2-month to half-year lifespan) [26, 27].

The system (Equation 1) can be rescaled in dimensionless form, given by a single parameter $R_{0}=\frac{A\;B}{\gamma \;\nu }$ (see Supplementary Material). Parameter $R{\;}_{0}$ measures the intensity of transmission in a hypothetical stationary Macdonald system. Namely, it has stable endemic state $\left(w^{*},y^{*}\right)>0$ when $R_{0}>1$, and a stable infection-free equilibrium ($w^{*}=y^{*}=0$) when $R_{0}<1$. The analysis in the current paper mostly employed the basic Macdonald system; however, several modifications are discussed in the Supplementary Material.

The nonstationary (seasonal) transmission environment, the subject of our present work, brings another important parameter—amplitude of seasonal variability [28]. Such seasonality can arise from multiple sources, that is varying snail populations or seasonally varying human behavior (water exposure/environmental contamination). Here, we focused on seasonal variation in snail population dynamics and its implications for sustained transmission and control. The potential influence of other variable inputs is discussed in the Supplementary Material.

### Incorporating a Seasonal Snail Environment

**Figure 1.**
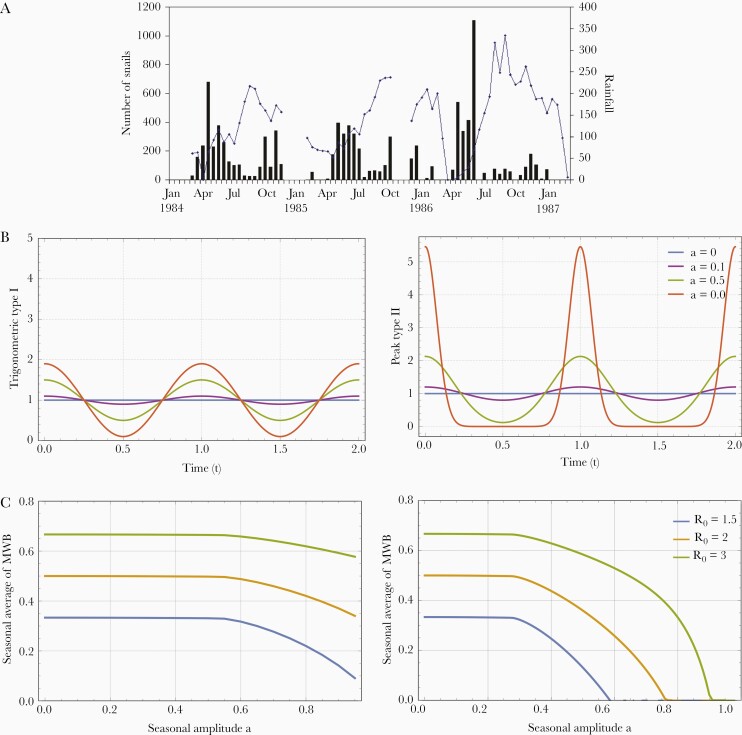
*A*, Rainfall data (solid bar) and snail population numbers collected in the Msambweni region of eastern Kenya from March 1984 to January 1987 [3, 22]. *B*, Examples of type-I (left) and type-II peak (right) seasonality with amplitude parameter a. The type-I seasonality was modeled by trigonometric function, 1+acos(2πt), and type-II seasonality was modeled by an elliptic theta function of amplitude 0≤a<1, $1+2\underset{n=1}{\overset{\infty }{\sum }}\;a^{n^{2}}\cos\left(2\pi \;n\;t\right)$. At small amplitude, a, both types are approximately equal, because higher-order Fourier modes become negligible. But as amplitude increases, they depart significantly in their variability (finite for type I and unlimited for type II). *C*, Seasonal average of mean worm burden (MWB), $\bar{w}\left(a\right)=\langle w^{*}\left(t,a\right)\rangle$ as a function of amplitude, a, for human-snail Macdonald systems Equation (4) for 3 values of basic reproduction number $R_{0}$ (transmission intensity), $R_{0}=1.5;2;3$. Left, results for a type-I trigonometric N(t,a) model; right, results for a type-II peak N(t,a) model. The curves indicate that for a lower $R_{0}$ and a higher seasonal amplitude, transmission becomes unsustainable in both type-I and type-II models. Not shown, these curves also depend on snail mortality, which in the case shown was ν=4.

Multiple environmental factors can affect snail population dynamics (reproduction, mortality/survival, development, and infection). They include temperature, rainfall (Figure 1A), food resources, agriculture, snail population predation, and environmental chemistry [3, 4, 19, 29–33]. We did not include all such factors explicitly, but considered their effects on snail dynamic in 2 ways: (1) a prescribed periodic snail population function N(t,a) of seasonal amplitude, a; and (2) a logistic snail population model driven by an underlying carrying capacity function K(t,a).

In both cases, the variability of *N* and *K* was determined by their amplitude parameter 0≤a<1, where a=0 corresponded to a stationary population value, while a larger a>0 marked the departure from this value. For our analysis, all population density functions were dimensionless, having been rescaled relative to a putative mean population density $N^{*}$.

The simplest mathematical form of periodic functions (*N, K*) is trigonometric,


$$\begin{array}{l} N\left(t,a\right)=1+a\;\;\cos\left(2\;\pi \;t\right),\;\;0\leq a\leq 1 \end{array}$$ (2)

referred to in this paper as type-I seasonality. The distinguishing features of type I are evenly distributed high and low seasons, where the snail population varies about its mean value (= 1) in equal proportions and evenly spaced across high and low seasons. This type-I system has limited range of seasonal variability (amplitude or variance).

However, in many cases, more extreme patterns of seasonality arise, for example, a short rainy season with high snail density, followed by longer dry periods of low density, or multiple seasonal peaks [22]. A convenient functional form for such variability can be described via an elliptic theta-function of amplitude (0≤a<1),


$$\begin{array}{l} N\left(t,a\right)=1+2\underset{n=1}{\overset{\infty }{\sum }}\;a^{n^{2}}\cos\left(2\pi \;n\;t\right) \end{array}$$ (3)

Such *N* becomes highly concentrated as a approaches 1 (Dirac delta-function) (Figure 1B). We refer to it as type-II (or peak) seasonality. The (nonlinear) amplitude of type-II (Equation 3) has different meaning than type I. Both types are approximately equal at small *a*, but as amplitude increases, they depart significantly in their variability. Data on snail abundance supports both patterns [4, 5, 17, 18, 22, 23]. Of course, they represent a crude approximation of such variability. Their simple analytic form is convenient for analysis and numeric simulation.

### The Macdonald Model System With Seasonal Snail Variation

The rescaled seasonal Macdonald system with a prescribed, variable snail population has the form


$$\begin{array}{l} \begin{array}{ll} & \frac{dw}{dt}=\gamma \left(y-w\right)\\ & \frac{dy}{dt}=\nu \left[R_{0}w\left(N\left(t,a\right)-y\right)-\mu \left(t\right)\;y\right] \end{array} \end{array}$$ (4)

These equations are derived similar to the stationary case, but with modifications to account for nonstationary snail density, N(t,a). Specifically, the infected snail prevalence variable 0<y(t)<1 of the stationary model is replaced by an infected snail density (0<y(t)<N(t,a)), and the constant snail mortality ν is replaced by time-dependent function, μ(t). (Details are provided in the Supplementary Material.)

For dynamic-snail population Macdonald system, Equation 4 is supplemented with a logistic snail-growth equation for variable N(t),


$$\begin{array}{l} \frac{dN}{dt}=\beta \left(1-N/K\right)N-\nu \;N \end{array}$$ (5)

Here β is maximal reproduction rate, K(t,a) is seasonal carrying capacity, and ν is natural snail mortality [22]. Snail dynamic (Equation 5) will automatically reproduce time-dependent mortality, μ(t).

The snail population in Equation 5 is thus decoupled from the standard Macdonald system of Equation 4. We note that snail infection typically has only a marginal effect on its overall population rates for reproduction/growth/mortality due to relatively low levels of patent (shedding) snails in natural environments [34, 35].

As withN(t,a), the periodic function K(t,a) can be either a trigonometric type (type I) or a peak type (type II). The logistic Equation 5 with periodic K(t,a) has a stable periodic solution $N^{*}\left(t,a\right)$, which plays the role of the prescribed function N(t,a) in the Macdonald case (Equation 4).

### Modeling Mass Drug Administration and Snail Control for the Revised Macdonald System

A single MDA session at time *T* in a host community with MWB w(t)was simulated in our model as an instantaneous reduction of MWB, $w\left(T\right)\to (1-\varepsilon_{H}\;)w\left(T\right)$, where the treatment effectiveness constant, $\varepsilon_{H}$, combines antiparasite drug efficacy (the fraction of killed worms), ε, and the population coverage fraction 0<f<1, that is $\varepsilon_{H}=\varepsilon f$. Such formulation implies each MDA session draws a random host pool (fraction *f*) for treatment. Another possibility is systematic noncompliance whereby the same population is excluded from treatment. The latter requires an extension of the Macdonald system discussed in Supplementary Material A16.

Instantaneous reduction of worm burden post MDA is due to short half-life (hours) of praziquantel relative to time scales of infection history (months to years). It is implemented via reinitialization of variable w(t) at time *T*. Snail control via molluscicide (at time *T*) was implemented in a similar fashion as an instantaneous event, whereby dynamic variables {N(t,a),y(t)} are dropped by factor $0<\varepsilon_{S}<1$ (reflecting the snail killing fraction), which depends on the efficacy of molluscicide application,


$$\begin{array}{l} N\left(T,a\right)\to (1-\varepsilon_{S})N\left(T,a\right);\;\;y\left(T\right)\to (1-\varepsilon_{S})y\left(T\right) \end{array}$$

## RESULTS

### Seasonal Snail Variability and its Effects on Endemicity of Human Infection

**Figure 2.**
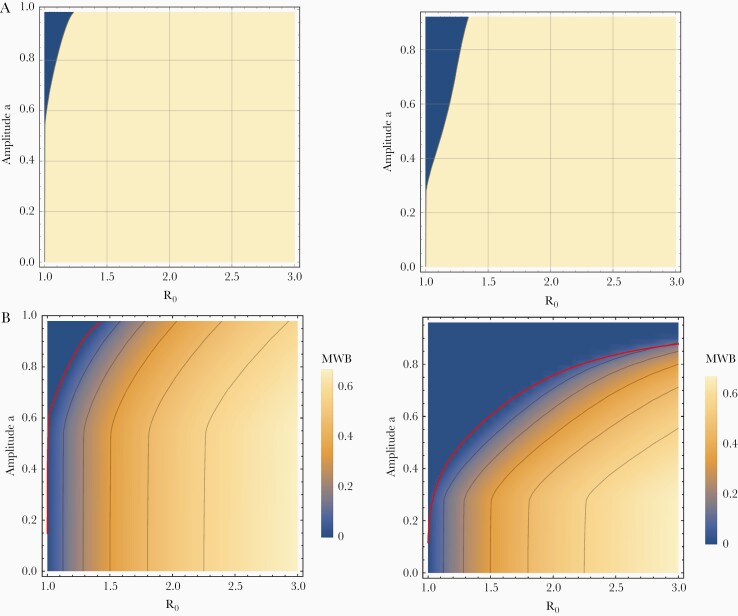
*A*, Parameter space analysis of Macdonald systems with seasonal snail population N(t,a) of type I (left), or type II (right) in the $\left(R_{0},a\right)$ plane. The blue shaded region corresponds to a stable infection-free equilibrium and is estimated from the stability analysis of the infection-free equilibrium (see Supplementary Material for details). In (*B*) a different procedure was used to define the stable endemic periodic solution $w\left(t,R_{0},a\right)$. The seasonal average of mean worm burden (MWB), $\bar{w}\left(R_{0},a\right)$, is computed numerically on a suitable grid. The red curve marks an approximated boundary between stable infection-free equilibrium and stable endemic periodic state. This boundary is not sharp (see Supplementary Figure 6 for details). The regions/isocontours in the figure depend on the snail mortality rate, taken here as ν=4.

The nonstationary Macdonald system with seasonally varying snail population, N(t,a), has 2 dimensionless parameters: $R_{0}$ (intensity of transmission) and amplitude (0≤a<1). We want to study their effect on sustainability of transmission. The role of stationary endemic equilibria is demonstrated here by time-periodic solutions$\left\{w^{*}\left(t,R_{0},a\right),y^{*}\left(t,R_{0},a\right)\right\}$. Their stability types and dependence on parameters $\left(R_{0},\;a\right)$ space is studied via seasonal average of the MWB-variable $w\left(t,R_{0},a\right)\to \bar{w}\left(a,R_{0}\right)$. The stationary case corresponds to (a=0). All results below are obtained via numeric simulations of the time-periodic dynamical Macdonald system. Key findings are listed below (see Supplementary Material for details):

1.
$\bar{w}\left(a,R_{0}\right)$ is a decreasing function of a, with maximum ${\bar{w}}_{\max}=1-1/R_{0}$, attained at a=0 (ie, stationary case), and minimal value at large values (a≈1) (Figure 1C). The drop of $\bar{w}\left(a,R_{0}\right)$ along the *a*-axis depends on the transmission intensity ($R_{0}$). It drops faster for smaller $R_{0}$, and can drop to 0, which means infection becomes unsustainable. Figure 1C shows functions $\bar{w}\left(a,R_{0}\right)$ for both types of seasonality and 3 values of $R_{0}$. For type I we observed a 15% drop of ($\bar{w}\left(1,R_{0}\right)/\bar{w}\left(0,R_{0}\right)$) for $R_{0}=3$, and a larger 35% drop and 75% drop for $R_{0}=2$ and $R_{0}=1.5$, respectively. This R_0_/amplitude effect was even more pronounced for type-II seasonality, where all periodic stable solutions become unsustainable at sufficiently large a.
2. The periodic Macdonald system (Equation 4) behaves qualitatively similar to a stationary Macdonald case (Equation 1). Specifically, it has infection-free equilibrium (w=0) and periodic endemic states ($w\left(t,R_{0},a\right)$), whose stability types are determined by parameters $\left(R_{0},a\right)$. Theoretically, the ($R_{0}$,a) space can be divided into 2 regions of possible long-term outcomes: a stable infection-free sector and a stable endemic state sector. But in practice, it is difficult to find the boundary numerically. In Figure 2 we show 2 types of parameter space analysis based on infection-free equilibrium and periodic endemic state, respectively. In both cases, increased seasonal amplitude makes infection less sustainable; transitioning from stable endemic to stable infection-free is gradual and difficult to assess computationally (see Supplementary Material for details).

In brief, this suggests that seasonal variability of snail numbers could make local transmission and persistence of infection potentially less sustainable and, thus, ultimately easier to control.

### Finding Optimal Timing for Control Interventions in a Seasonal Transmission Environment

#### Optimal MDA Timing

We next explored the impacts of 2 different types of control interventions, MDA and molluscicide. The former has no direct effect on snail populations, while the latter targets snails specifically. This analysis required use of the dynamic snail population model, that is the coupled system of Equations 4 and 5. The key input here was the seasonal carrying capacity function, K(t,a), represented by the type-I or type-II periodic functions.

In all cases, we used the endemic periodic state $\left\{w^{*}\left(t,a\right),y^{*}\left(t,a\right)\right\}$ to initialize the system, and ran a 6-year control program, as suggested by the World Health Organization (WHO) [14, 36]. Specifically, annual MDA has an effective parameter $\varepsilon_{H}=\varepsilon \;f$, which combines drug efficacy (ε=0.70−0.85) and the population coverage fraction (f). Outcomes of such intervention depend foremost on transmission intensity, $R_{0}$. Here we took an intermediate value ($R_{0}=3$) [36], and strong seasonality, amplitude a=0.9 for type I, and a=0.6for type II. Other examples are discussed in Supplementary Figures 12–17).

**Figure 3.**
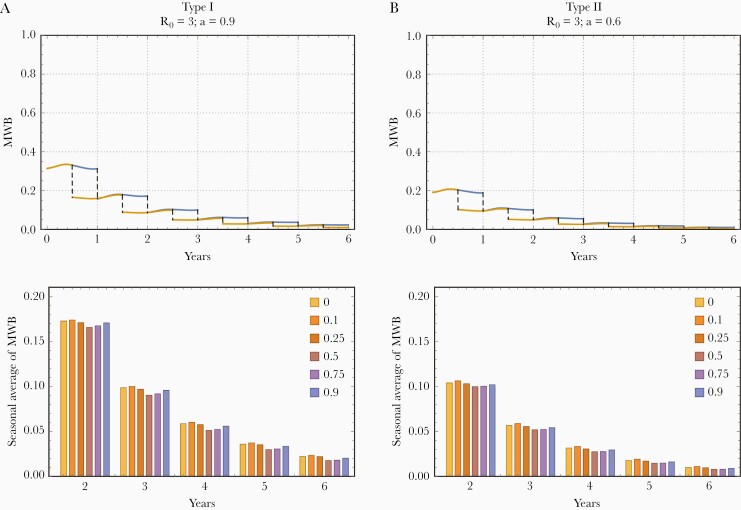
Effect of annual mass drug administration (MDA) on seasonal mean worm burden (MWB) patterns for 2 types of seasonality at high transmission environment and strong seasonal variability. A 6-year control program was run for Macdonald-type systems having dynamic snail populations (ie, seasonal carrying capacity functions K(t,a) of trigonometric type I [left] or peak type II [right]). Here we used the MDA efficacy $\varepsilon_{H}=50\%$, corresponding to drug efficacy (70%–85% reduction in MWB) combined with the MDA treatment population coverage fraction (60%–70%). The upper panels show 6-year histories with MDA given at the start of each season τ=0 (blue), or at midseason τ=1/2 (yellow). The lower panels show the 5-year seasonal averages for MWB among local humans when different seasonal timings (0≤τ<1) were used for implementation. For each seasonal timing, $\tau =\left\{0,\frac{1}{10},\frac{1}{4},\frac{1}{2},\frac{3}{4},\frac{9}{10}\right\}$, we averaged MWB over the proper 1-year time interval, ie, over the period (τ,τ+1), for successive 6 years. Qualitatively, these results look similar to $R_{0}=6$ (high transmission intensity) case discussed in Supplementary Figure 14, but the seasonal difference is less significant.

We found that different choices for intraseasonal MDA timing during the annual cycle (time =0≤τ<1) can affect the MWB reduction. The upper panels of Figure 3 demonstrate this effect by comparing MDA histories implemented at τ=0 (the season’s start, set up at the peak of snail population) versus τ=0.5 (midseason). To estimate the optimal MDA timing, that is the greatest MWB reduction by year 6, we ran multiple simulations with different choices (0<τ<1). In each case, we averaged the resulting MWB-solution {w(t)} over the intertreatment time interval, [τ,τ+1].

In the lower panels of Figure 3, we show the resulting average MWB values over a 6-year control history for different choices of MDA implementation time, τ. The difference between the “best” and “worst” timing, was marginal—a 2.5% MWB reduction by year 6. Such an effect, however, could be more significant in areas with high transmission intensity, for example, $R_{0}=6$ (Supplementary Table 4). The optimal timing for MDA administration varied in the range, 1/2<τ<3/4, that is close to the midseason for high amplitude (a) but shifted toward τ≈3/4 for moderate *a*. The pattern was similar for both types of seasonality. The results are summarized in Table 1, which shows, for each τ tested, the post intervention average MWB ($\bar{w}\left(Y_{6},\tau \right)/\bar{w}\left(Y_{0},\tau \right)$) endemic values.

**Table 1.** Effect of Annual Mass Drug Administration (MDA) in Terms of Mean Worm Burden (MWB) Percent Reduction Among Local Human Populations

Seasonality	MDA Efficacy $\varepsilon_{H}$ ,%	Time of MDA, Fraction of Seasonal Cycle					
		0	1/10	1/4	1/2	3/4	9/10
Type I							
Strong seasonality amplitude a=0.9	50	87.1	86.5	82.7	**89.3**	89.2	88.1
	55	90.6	90.0	90.6	**92.5**	92.5	91.5
	70	97.1	96.8	97.1	**98.1**	98.2	97.7
Moderate seasonality amplitude a=0.5	50	82.8	82.3	82.2	83.6	**84.1**	83.4
	55	86.9	86.3	86.2	87.6	**88.2**	87.5
	70	95.3	94.9	94.8	95.7	**96.2**	95.8
Type II							
Strong seasonality amplitude a=0.6	50	90.3	89.6	90.7	**92.7**	92.0	91.3
	55	93.2	92.5	93.5	**94.7**	94.6	94.0
	70	98.1	97.8	98.3	**98.7**	98.8	98.6
Moderate seasonality amplitude a=0.3	50	82.9	82.3	82.2	84.0	**84.6**	83.7
	55	87.0	86.4	86.3	87.9	**88.5**	87.8
	70	95.4	94.9	94.7	95.9	**96.4**	95.9

Data are percent reduction in MWB.

We simulated a 6-year MDA regimen for a Macdonald-type model system having dynamic snail populations with seasonal carrying capacity function K(t,a) of trigonometric types I or peak type II. Impact was measured in terms of relative MWB reduction for local residents (year 6 over endemic year 0) using seasonal average values $\bar{w}\left(Y_{6},\tau \right)/\bar{w}\left(Y_{0},\tau \right)$ of MWB. We examined several different values of intraseasonal timing for MDA, $\tau =\left\{0,\frac{1}{10},\frac{1}{4},\frac{1}{2},\frac{3}{4},\frac{9}{10}\right\}$, as fractions of the seasonal cycles, 3 possible levels of MDA efficacy (instantaneous reduction of mean worm burden) $\varepsilon_{H}=\left\{50\%,55\%,70\%\right\}$ as the combined effect of drug efficacy (fraction of killed worms) and population coverage fraction, and 2 values of seasonal amplitude, a. The optimal timing (highlighted in bold) varied in the range ${}^{1}╱{}_{2}<\tau <{}^{3}╱{}_{4}\;$. The optimum was close to mid-season $\tau \approx {}^{1}╱{}_{2}\;$ in the presence of strong seasonal amplitude (a) and shifted toward $\tau \approx {}^{3}╱{}_{4}\;$ at moderate seasonal amplitude (a).

#### Optimal Timing for Molluscicide Application and for Integrated Control Strategies

We next used our dynamic snail models to estimate the optimal timing for molluscicide-based snail control, and for an integrated strategy of MDA plus snail control.

**Figure 4.**
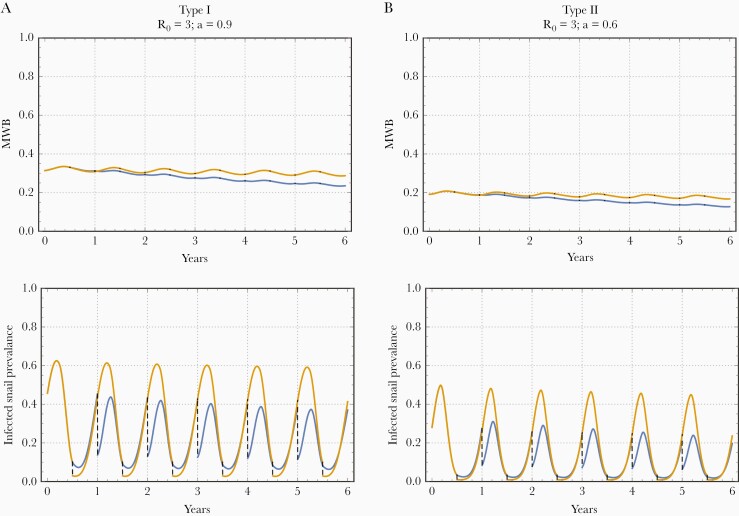
Effect of molluscicide timing on transmission dynamics of Macdonald-type systems with seasonal snail populations having carrying capacity K(t,a) of trigonometric type I (left) or peak type II (right) in a high-transmission environment and strong seasonal variability. Here we used molluscicide efficacy (percent of killed snails) $\varepsilon_{S}=70\%.$ The 2 colors correspond to different seasonal timing of molluscicide application: at the start of the season, τ=0 (blue) or at midseason, $\tau ={}^{1}╱{}_{2}\;$ (yellow). The effect of seasonal timing on long-term patterns of transmission (6-year history) can be significant: τ=0 implementation ultimately gives higher worm-burden reduction among local humans than does $\tau ={}^{1}╱{}_{2}\;$ implementation. Qualitatively, these results look similar to $R_{0}=6$ (high transmission intensity) case discussed in Supplementary Figure 15, but the seasonal difference is less significant.

We used the same $R_{0}$ and *a* values as above. Snail mortality and maximal growth were fixed at ν=4/year, β=20/year, broadly consistent with some estimated parameters [22]. Figure 4 shows a projected 6-year history for a molluscicide-only control program, comparing type-I and type-II seasonality simulations. Two choices of molluscicide timing are compared, τ=0 (blue) and τ=1/2 (yellow). This effect of molluscicide on human infection (8%–38% reduction in MWB; Table 2) was less significant than drug treatment (82%–98% reduction in MWB; Table 1). Nevertheless, if snail control was accompanied by introduction of optimally timed MDA, human MWB (Table 3) and infected snail density y(t) (not shown) have approached near-elimination state after a 6-year program.

**Table 2.** Effect of Annual Molluscicide Application in Terms of Mean Worm Burden (MWB) Reduction in the Human Host Population

Seasonality	Molluscicide Efficacy$\varepsilon_{S}$ ,%	Time Points Within the Seasonal Cycle for Molluscicide Application τ					
		0	1/10	1/4	1/2	3/4	9/10
Type I							
Strong seasonality amplitude a=0.9	50	**13.5**	13.3	8.0	3.8	8.4	11.9
	70	**20.3**	19.4	11.9	5.7	13.5	18.3
	90	**30.7**	28.2	17.1	10.6	23.8	29.4
Moderate seasonality amplitude a=0.5	50	**8.8**	8.7	7.5	4.8	6.4	8.1
	70	**13.7**	13.6	11.3	7.8	10.3	12.8
	90	**22.0**	21.1	17.0	12.3	17.8	21.2
Type II							
Strong seasonality amplitude a=0.6	50	**19.2**	18.0	9.2	6.5	13.2	17.2
	70	**27.0**	24.9	12.8	8.5	19.6	24.8
	90	**38.0**	33.9	17.6	14.2	32.0	37.1
Moderate seasonality amplitude a=0.3	50	**9.3**	9.2	7.7	4.5	6.4	8.4
	70	**14.5**	14.3	11.5	7.0	10.4	13.4
	90	**23.1**	22.1	17.2	11.6	18.3	22.2

Data are percent reduction in MWB.

A 6-year control program was simulated using a Macdonald-type model system having a dynamic snail population seasonality with carrying capacity function K(t,a) of trigonometric type I or peak type II structure. As for Table 1, progress was measured by relative seasonal average reduction of MWB $\bar{w}\left(Y_{6},\tau \right)/\bar{w}\left(Y_{0},\tau \right)$. We examined several possible intraseasonal timings for molluscicide application,$\tau =\left\{0,\frac{1}{10},\frac{1}{4},\frac{1}{2},\frac{3}{4},\frac{9}{10}\right\}$, as fractions of the seasonal cycle, 3 possible levels of molluscicide efficacy (percent of killed snails) $\varepsilon_{S}=\left\{50\%,70\%,90\%\right\}$, and 2 choices of the seasonal amplitude parameter (moderate or high). Optimal timing for molluscicide control in all cases was τ=0 (highlighted in bold), ie, at the start/end of the season, when the snail population or its carrying capacity reached its maximum.

**Table 3.** Comparison of Optimal-Control Progress for Mass Drug Administration (MDA) Alone Versus an Integrated MDA Plus Molluscicide-Based Snail Control Strategy

MDA Efficacy $\varepsilon_{H}$, %	MDA Only	MDA + Snail Control εs, %			MDA Only	MDA + Snail Control εs,%		
		50	70	90		50	70	90
Type I								
	Strong seasonality amplitude a=0.9				Moderate seasonality amplitude a=0.5			
50	89.3	95.7	96.4	97.2	84.1	92.7	93.8	95.3
55	92.5	97.3	97.8	98.3	88.2	95.1	95.9	96.9
70	98.1	99.5	99.6	99.7	96.2	98.9	99.1	99.4
Type II								
	Strong seasonality amplitude a=0.6				Moderate seasonality amplitude a=0.3			
50	92.7	99.7	99.8	99.9	84.6	98.7	99.1	99.5
55	94.7	99.9	99.9	100.0	88.5	99.4	99.6	99.8
70	98.7	100.0	100.0	100.0	96.4	99.9	100.0	100.0

Data are percent reduction in mean worm burden (MWB).

As in Tables 1 and 2, progress was measured by relative seasonal average reduction of MWB $\bar{w}\left(Y_{6},\tau \right)/\bar{w}\left(Y_{0},\tau \right)$. A 6-year control program was simulated for a Macdonald-type model system having dynamic snail populations (K(t,a) of type-I or type-II), 3 levels of MDA efficacy $\varepsilon_{H}=\left\{50\%,55\%,70\%\right\}$, 3 levels of molluscicide efficacy $\varepsilon_{S}=\left\{50\%,70\%,90\%\right\}$, and 2 different amplitudes of seasonality. The impact for MDA-only was estimated at its optimal time of delivery, as suggested by Table 1. The impact of the MDA + snail strategy was based on separate delivery at their individual optimal timings, τ, as suggested by Table 1 and Table 2, namely $\tau ={}^{1}╱{}_{2}\;or{}^{3}╱{}_{4}\;$ for MDA, and τ=0 for molluscicide application.

The optimal timing of molluscicide implementation for maximal MWB reduction (Table 2) fell near the start of the season (τ≈0) where carrying capacity function, K(t,a), and snail population density, N(t,a), approach maximal values. The overall progress measured as above by $\bar{w}\left(Y_{6},\tau \right)/\bar{w}\left(Y_{0},\tau \right)$ varied in the range 8%–38%, depending on the type and amplitude of seasonality and the efficiency, $\varepsilon_{S}$, of molluscicide. We also observed that the effect of optimal timing was much more significant for molluscicide than for MDA (Table 2).

A combined MDA plus molluscicide strategy was next simulated for 3 choices of molluscicide efficacy, $\varepsilon_{S}=\left\{50\%,70\%,90\%\right\}$, and 3 choices for MDA efficacy, $\varepsilon_{H}=\left\{50\%,55\%,70\%\right\}$. For this analysis, each intervention was to be given at its own optimal timing, τ, as suggested by the results in Table 1 and Table 2, namely τ=0.5−0.75 for MDA and τ=0 for molluscicide. We then compared 2 optimized strategies, MDA alone versus MDA plus molluscicide in Table 3. This indicates that integrated control can bring a significant improvement above an MDA-only strategy when each intervention is properly timed. Depending on seasonal amplitude, the type of seasonality, transmission intensity $R_{0}$, molluscicide clearing efficiency $\varepsilon_{S}$, and MDA efficacy$\varepsilon_{H}$, the year 6 worm burden could be brought to elimination, relative to the baseline (endemic) values.

## DISCUSSION

Conventional modeling approaches to infection transmission dynamics in a seasonally varying environment often employ time-averaged stationary models [15]. Such approximation can be justified when seasonal variability is low relative to the mean state but may not hold in general. Here, we studied the effects of seasonal snail population using Macdonald-type model with 2 basic patterns of variability, trigonometric (type I), and peak (type II). Both have an explicit mathematical form, but our analysis has employed primarily numeric simulations of the appropriate dynamical systems.

The key inputs for our analysis are dimensionless parameters: (1) the basic reproduction number of the stationary (seasonal-mean) system, $R_{0}$ (intensity of transmission); and (2) the amplitude (a) of seasonal snail variability. We addressed several questions of the combined effect of $\left(R_{0},a\right)$ on periodic and stationary patterns of human and snail infection levels, and their seasonal averages, exemplified by MWB function $\bar{w}\left(R_{0},a\right)$. As function of amplitude, a, it typically maintained a plateau region at small or moderate a values, so seasonal averaging for Macdonald system would still be approximately valid. However, it would fail for large amplitude, where $\bar{w}\left(R_{0},a\right)$ underwent a rapid decay at high amplitude values, in some cases to $\bar{w}=0$(elimination). Along these lines, we identified specific parameter ranges where transmission becomes unsustainable. Overall, we found increased seasonality makes infection transmission and endemicity less sustainable, hence more amenable to control interventions, as compared to areas with unchanging (stationary) annual transmission. Future studies could explore whether such conclusions could hold for other environmentally and seasonally mediated infectious diseases, like malaria. Most of our analysis was done using the basic Macdonald system, but some modifications were extended to evaluate other transmission models, including a Macdonald system with worm mating [25] and the stratified worm burden system [37, 38] with comparable results (see Supplementary Material for details).

Seasonality can affect snail population dynamics and human behavior (environmental exposure and contact rates). The 2 factors are sometimes combined into a single seasonal transmission rate [16, 39]. There are, however, significant differences between them (Supplementary Material), and the current analysis has specifically focused on seasonal snail dynamics.

We have applied our models to leverage seasonality for the optimal timing of repeated control interventions (including annual MDA, molluscicide application, or integrated intervention strategies that combine the 2 approaches) to predict which approach could achieve maximal reductions in parasite burden over a limited time span (6–10 years). The optimal intraseasonal timing (0<τ<1) varied for different interventions; the season start was defined by maximal snail density. For MDA alone, we found the optimal timing to be in the range, 1/2<τ<3/4, near the minimal snail density (or its carrying capacity).

For molluscicide-based control, the optimal timing of delivery was closer to the start of season, that is at the snail population peak. The overall effect of mollusciciding on human worm burden reduction was less significant than MDA at short duration, even at high snail killing efficacy. But the effect of optimal timing (τ) was more pronounced for molluscicide than for MDA. The combined strategy (MDA plus molluscicide), with proper timing for each, was found to provide an enhanced reduction of MWB compared to MDA alone. This suggests that addition of snail control can add substantially to the program impact, in particular at high transmission intensity where MDA alone may not be sufficient (Supplementary Tables 4–6).

In general, control outcomes depend on transmission intensity, strength and patterns of seasonality, drug and molluscicide efficacy, and control duration. A 6-year program is an intermediate-range based on WHO guidelines [14, 36]. In some cases, it could lead to near-elimination after a 6-year period. In other cases, additional treatment or an integrated strategy combining MDA with molluscicide is needed. These results are consistent with the results from earlier modeling papers [36, 40]. Another important factor is the simulation of the MDA delivery for a prescribed population fraction. For example, it could be a randomly drawn pool of residents or involve a systematically noncompliant pool. We observed that systematic noncompliance could significantly slow the progress of MDA program (see Supplementary Material for details). Identifying robust and optimal combination strategies for schistosomiasis control under these various conditions is paramount for achieving and maintaining WHO schistosomiasis elimination goals. Future work should include the development of systematic optimization procedures for identifying optimal control interventions across transmission settings.

We believe the basic conclusions on seasonal timing are robust and would hold in different environments, for different transmission intensity, $R_{0}$, and for different schedules of MDA delivery. Furthermore, we expect they would hold for other transmission models, and with more realistic patterns of snail population dynamics driven by temperature and rainfall data [3, 22, 35, 41].

## Supplementary Data

Supplementary materials are available at *The Journal of Infectious Diseases* online. Consisting of data provided by the authors to benefit the reader, the posted materials are not copyedited and are the sole responsibility of the authors, so questions or comments should be addressed to the corresponding author.

jiaa746_suppl_Supplementary_Information

## Notes

***Author contributions.*** Q. H., D. G., and C. H. K. designed research; Q. H. and D. G. performed research; Q. H., D. G., M. N. M., and C. H. K. analyzed models; and all authors interpreted the results and contributed to writing.

***Acknowledgment.*** We would like to thank the reviewers and handling editor for their helpful comments and suggestions, which improved the presentation of the manuscript.

***Disclaimer.*** The funders had no role in the design and conduct of the study, collection, management, analysis and interpretation of the data, preparation, review, or approval of the manuscript, or the decision to submit the manuscript for publication.

***Financial support.*** This work was supported by the Schistosomiasis Consortium for Operational Research and Evaluation funded by the University of Georgia Research Foundation through a grant from the Bill and Melinda Gates Foundation (grant number OPP50816 to D. G. and C. H. K.).

***Potential conflicts of interest.*** All authors: No reported conflicts of interest. All authors have submitted the ICMJE Form for Disclosure of Potential Conflicts of Interest. Conflicts that the editors consider relevant to the content of the manuscript have been disclosed.
