# Supplementary material for: Schistosoma Transmission in a Dynamic Seasonal Environment and its Impact on the Effectiveness of Disease Control
Source: J Infect Dis. 2020 Dec 2;225(6):1050–61. doi: 10.1093/infdis/jiaa746 (PMC8921996; doi:10.1093/infdis/jiaa746)
Supplement: jiaa746_suppl_Supplementary_Information [file jiaa746_suppl_supplementary_information.docx]

Supplementary Information for

***Schistosoma* transmission in a dynamic seasonal environment and its impact on the effectiveness of disease control**

Qimin Huang, David Gurarie, Martial Ndeffo-Mbah, Emily Li, Charles H. King

**This PDF file includes:**

Appendix A: transmission models

Appendix B: results

Figures S1 to S18

Tables S1-S6

SI References

Appendix A: transmission models

1. **The rescaled MacDonald system** consists of human mean worm burden (MWB) equation, for a dimensionless MWB variable$\left\{w\to\frac{\gamma}{A}w\right\}$ , and snail prevalence y,$0<\left(w,y\right)<1$ .

$$\begin{matrix} \frac{dw}{dt}=\gamma\left(y-w\right) \\ \frac{dy}{dt}=\nu\left[R_{0}w\left(1-y\right)-y\right] \end{matrix}$$

Equilibria and dynamic patterns of depend on a single dimensionless BRN parameter$R_{0}=\frac{A B}{\gamma\nu}$ , where transmission coefficients$(A,B)$ are proportional to population densities ($A\propto N$- snail, $B\propto H$- human), $\gamma$ - worm mortality + host turnover, $\nu$ -snail mortality. In a stationary environment, such a system has stable endemic equilibrium, $w^{*}=y^{*}=1-1/R_{0}$, and an infection-free equilibrium $w^{*}=y^{*}=0$. Their stability types depend on $R_{0}$, $R_{0}>1$ gives a stable endemic state and an unstable infection-free equilibrium, while $R_{0}<1$ would move “endemic” into unphysical range and make the “infection-free” state stable.

We shall discuss two modifications of the basic system .

**The Reduced MacDonald system** is derived by replacing the dynamic y-variable with a quasi-equilibrium value, $y\approx\frac{R_{0}w}{1+R_{0}w}$from the snail equation , hence, a single worm equation -

$$\frac{dw}{dt}=\left(\frac{R_{0}}{1+R_{0}w}-1\right)w$$

Such reduction can be justified by much faster snail turnover rate compared to worm mortality and human turnover ($\gamma/\nu\ll1$ - small parameter).

**The MacDonald model system with worm mating and aggregation.**  Many conventional approaches, going back to the original MacDonald paper [1], assume worm burden is distributed in host population according to a negative binomial (NB) pattern with mean value $w\left(t\right)$, and aggregation parameter $k>0$ ([2-4, 5 ]). In such a setting, the force of snail infection is no longer proportional to MWB $w\left(t\right)$. Instead, one should use mean mated count burden (MCB), which depends on worm distribution within the human host community and the worms’ mating behavior. For an assumed NB – distribution of worm burden, MCB can be estimated via a so-called mating function , namely MCB = .

When worm mating and aggregation are added to the system’s dynamics, the simple MWB factor in the snail FOI (force of infection) is replaced by the mating function: , which depends on worm distribution in host population, and mated count of different worm strata (see, e.g., [6, 7]),

The **rescaled** MacDonald system with worm mating and aggregation is:

The **reduced** MacDonald system with worm mating takes the form**:**

Compared to the simple MacDonald model, system depends on 2 dimensionless parameters, transmission coefficients $\left(A/\gamma; B/\nu\right)$ , or pair $\left(R_{0}=AB/\gamma\nu; A/\gamma\right)$, rather than single $R_{0}$. There are other fundamental differences between simple MacDonald, and its modified version. The former has two equilibria (infection-free and endemic), whose stability types are determined by $R_{0}$ ($R_{0}>1$- stable endemic, $R_{0}<1$-stable infection-free), i.e., a *saddle-no*de type. The modified system is *bistable* for sufficiently large $R_{0}>R_{c}>1$ , above critical value $R_{c}$. Fig. S9 illustrates a critical $R_{c}=2.4$, for one specific choice of transmission coefficients. It has stable equilibria (infection-free + endemic), and an intermediate (unstable) breakpoint. Such breakpoints have important implications for MDA control, explained below.

**Periodic seasonal forcing** is determined by relative snail population density $N\left(t,a\right)$ with mean value , using either a trigonometric function$N\left(t,a\right)=1+a \cos\left(2\pi t\right)$ of amplitude ($0\leq a<1$) (termed here as type I), or a ‘single-peak’ type function $N\left(t,a\right)$, termed type II. Some modifications are needed to accommodate such variable snail population, namely (i) the prevalence variable $y\left(t\right)$ is replaced by infected snail density ($0<y\left(t\right)<N\left(t,a\right)$),(ii) fixed snail mortality in , is replaced by a time-dependent rate function

$$\mu\left(t\right)=\max\left\{1, -\frac{N'\left(t,a\right)}{\nu N\left(t,a\right)}\right\}$$

The latter is important during dry season (decaying population$N\left(t,a\right)$), when its loss rate is faster than the natural mortality (under normal conditions - abundant food supply). The resulting

**Rescaled seasonal equations** take the form:

$$\begin{matrix} \frac{dw}{dt}=\gamma\left(y-w\right) \\ \frac{dy}{dt}=\nu\left[R_{0}w\left(N\left(t,a\right)-y\right)-\mu\left(t\right) y\right] \end{matrix}$$

Its dynamic patterns and equilibria depend on two parameters $\left(R_{0},a\right)$.

**Reduced seasonal equations** are given by

$$\frac{dw}{dt}=\left(\frac{R_{0} N\left(t,a\right)}{\mu\left(t\right)+R_{0}w}-1\right)w$$

In either case, the full (2D) system or the reduced 1D system, there exists a stable periodic solution $w^{*}\left(t\right)$, which plays the role of stable endemic equilibrium. In can be computed from the fixed point of the corresponding Poincare (period) map.

**Dynamic human-snail contacts in MacDonald system**. Seasonality can affect both snail population biology, and human-snail contacts. The corresponding seasonal-contact MacDonald system (assuming a stationary snail process) is given by

$$\begin{matrix} \frac{dw}{dt}=\omega\left(t\right) y-w \\ \frac{dy}{dt}=\frac{\nu}{\gamma}\left[R_{0} \omega\left(t\right) w\left(1-y\right)-y\right] \end{matrix}$$

A qualitative parameter-space analysis of was given in [8] . While two systems - bear some similarity (periodic transmission coefficients) the analysis and outcomes are quite different.

**The Stratified Worm Burden (SWB) modeling approach** was developed in several papers ([9-11]). The basic variables in SWB are worm burden strata $\left\{h_{m}\left(t\right)\right\}$ - population fractions that carry ($m=0,1,2,...$) adult worms. The MacDonald-type Mean Worm Burden (MWB) system can be derived from SWB, where variable $w\left(t\right)=\sum_{m=0}^{\infty} m h_{m}\left(t\right)$ -mean values of the burden distribution in human hosts. The SWB has many advantages compared to reduced (MacDonald-type) models. It carries a detailed information on burden distribution and egg release by mated worm couples in host populations, with no prior assumptions. Within-host worm biology (mating, mortality, fecundity) is naturally accommodated in SWB. While SWB is a multi-variable system, it has the same parameters as “small” (2D) MacDonald. The main difference between MWB and SWB comes in the calculation of the snail force of infection (FOI), $\Lambda$. For the MacDonald system, $\Lambda$is a function of a single (MWB) variable, *w*. For the SWB, it depends on human infectivity E – the combined egg release by all human worm burden strata, as a function of worm mating and fecundity. Thus, for the SWB model, there is no direct link between w and E.

SWB equilibria and dynamic patterns are closer to simple MacDonald system with stable/unstable pair of equilibria (“infection-free” , “endemic”) rather than a bistable ‘MacDonald system with mating’ (stable “infection-free” / “endemic” states, along with intermediate “breakpoint”) (see [7, 10]).

**Basic reproduction number $R_{0}$of a coupled SWB-snail model.** Here we use SWB human equations, coupled to an extended SEI- snail model with dynamic (logistic) population growth. Our goal is to derive the$R_{0}$-formula for such a system. We call the SEI snail-population variables: *x*- susceptible; *y*- exposed (prepatent); *z*- infected (patent), and total snail density $N=x+y+z$. They obey a coupled differential (ODE) system

$$\begin{matrix} \frac{dx}{dt}=\beta\left(1-\frac{N}{K}\right)x-\Lambda x-\nu x \\ \frac{dy}{dt}=\Lambda x-\left(r+\nu\right) y \\ \frac{dz}{dt}=c r y-\nu z \end{matrix}$$

The force of snail infection (FOI) $\Lambda\left(E\right)$ is a function of human infectivity *E*, proportional to MWB$w\left(t\right)$for MacDonald systems, or to ‘mean mated count’ for SWB. Conventional approaches use simple linear$\Lambda\left(E\right)$, like , , but in paper [9] we derived a nonlinear FOI.

In either case, the relevant input parameters are listed below in Table A1.

**Table A1**: Input parameters for the coupled SWB-snail model

| $\Delta w>1$ | SWB worm step (threshold for mating) |
| --- | --- |
| $\rho_{0}$ | Mean egg-release/mated female worm |
|  | Mean mated couple count for m-stratum (m adult worms) |
| $\gamma,\mu$ | Worm mortality and host population turnover |
| *a* | Probability of worm establishment/contact /snail |
| $\omega$ | Human-snail contact rate |
| *b* | Probability of snail invasion by miracidia |
| *N* | Snail density/unit habitat |
| *H* | human population density/unit habitat |
| $\nu$ | Snail mortality |
| $r$ | Patency conversion rate |
| $\beta$ | Maximal growth rate (for logistic snail population) |
| $A=a \omega N$ $B=b \omega H \rho_{0}$ | Transmission coefficients: snail-to-human and human-to-snail |
| $\frac{1-\nu/\beta}{1+\nu/\beta}$ | Snail contribution to BRN of the coupled system |

The$R_{0}$of the coupled SWB-snail system consists of several factors: (i) the conventional MacDonald $R_{0}$, expressed through transmission coefficients $A; B$ and worm, snail, host mortality/turnover rates; (ii) the SWB factor; and (iii) snail factors.

$$R_{0}=\frac{A B}{2\left(\gamma+\mu\right)\nu} \left[1-2^{-\Delta w}\left(\begin{matrix} \Delta w \\ \Delta w/2 \end{matrix}\right)\right] \frac{\left(1-\nu/\beta\right)}{\left(1+\nu/r\right)}$$

This formula can be extended to demographically structured host populations (e.g. children + adults). Formula (A11) can be derived from the Jacobian analysis of the coupled system at infection-free equilibrium.

As in the simple MacDonald system, the SWB $R_{0}$can distinguish between stable endemic state ($R_{0}^{SWB}>R_{c}$ - critical level), and stable “infection-free” ($R_{0}^{SWB}<R_{c}$). But unlike the MacDonald system, it carries no information on the endemic state or the impact of dynamic interventions, e.g. long-term MDA outcomes.

In fact, more detailed information of SWB equilibria can be derived from two transmission coefficients, A and B, rather than their product (as in the $R_{0}$formulation). As such, communities with identical levels of human infection but different snail environments (hence different pair values for A and B) can exhibit vastly divergent control outcomes despite having nominally identical R_0_ values.

Appendix B: Results

### Analysis of equilibria and relaxation

The basic MacDonald model for *Schistosoma* transmission combines human and snail components of the two-host community ecology, represented by the mean worm burden (MWB)$w\left(t\right)$ among humans, and by a local infected snail prevalence (or density) function,$y\left(t\right)$. The key dimensionless parameter for analysis of such system in a stationary state case is the basic reproduction number,$R_{0}$. For nonstationary (periodic) systems, e.g., with a varying seasonal snail density $N\left(t,a\right)$, there are two essential inputs, $R_{0}$(seasonal-mean intensity of transmission), and amplitude ($1\leq a<1$) of the seasonal (snail) variability. We considered two types of seasonal snail dynamics: type I (trigonometric function $N\left(t,a\right)=1+a \cos\left(2\pi t\right)$and type II (peak seasonality), given by the elliptic theta function. Typical solution curves $\left\{K\left(t,a\right),N\left(t,a\right),y\left(t\right)\right\}$for type I-II seasonality are shown in Fig. S1. In our setup carrying capacity (CC) function $K\left(t,a\right)$ attains its maximal and minimal values at $t=0$ and $t=1/2$, while population $N\left(t,a\right)$ lags behind by about 0.1 of season, followed by$y\left(t\right)$.

The role of endemic equilibria in nonstationary MacDonald systems is played by time-periodic solutions $w^{*}\left(t,R_{0},a\right)$. We examined these “periodic equilibria”, their stability, and their persistence in $\left(R_{0}, a\right)$parameter space. The stationary case would correspond to $a=0$. The key conclusions of our analysis are listed below:

1. The stationary 2D MacDonald system for MWB variable $w\left(t\right)$ and infected snail prevalence $y\left(t\right)$, and its reduced version (single MWB) share the same equilibria and $R_{0}$, but when perturbed from equilibrium (e.g. via mass drug administration (MDA) for parasite treatment and control) their relaxation patterns back to equilibrium will differ, with the reduced system relaxing faster than the 2D system (Fig. S2). The faster relaxation rate (rebound) would suggest a less efficient MDA impact for control in reduced system model than that predicted by the full MacDonald host-vector system model.
2. The role of stationary equilibria in a variable seasonal environment is played by periodic (dynamic) solutions$\left\{w\left(t,a\right),y\left(t,a\right)\right\}$, shown in Figs. S3-S4.
3. For type I seasonality, the reduced (single-host MWB) model maintains a similar qualitative pattern, where function $\bar{w}\left(a\right)$ decays with $a$. But its sustained levels and seasonal mean MWB values are higher than the full 2D MacDonald system (Fig. S3 and Fig. S5). Seasonal variability is relatively low for the human worm burden function $w^{*}\left(t\right)$ (<5%), but there is much higher variation for infected snail density $y^{*}\left(t\right)$. Peak worm burden $w^{*}\left(t\right)$ lags behind peak snail density $N(t)$ (and infected snail density $y(t)$) by about one quarter of a season.
4. For type II seasonality, increased $a$ (restricted wet season) tended to lower periodic equilibria. As above, we observed marked difference between the periodic patterns of reduced vs. complete MacDonald system (Figs. S4-S5). Their seasonal mean functions $\bar{w}\left(a,R_{0}\right)$ also departed significantly, particularly at large a, with the complete 2D MacDonald predicting elimination at sufficiently high $a$, while reduced model maintaining ‘positive’ endemic value for all $a$.
5. **Stability regions.** By analogy with the stationary case, we ask for regions of stability ‘zero equilibrium’ vs. ‘periodic state’, in the $\left(R_{0},a\right)$ parameter space. In the stationary case, two regions are strictly delineated by basic reproduction number: $R_{0}<1$ - stable infection-free equilibrium vs. $R_{0}>1$- stable endemic. The Periodic MacDonald system has no analytic solution of the stability problem. We employ numeric simulations by scanning parameter space on a suitable $\left(R_{0},a\right)$ - grid. Different approaches are used for two dynamic states of the system. For stable infection-free equilibrium, we linearize MacDonald system at infection-free equilibrium, and study the resulting periodic Jacobian matrix-function $J\left(t,R_{0},a\right)$ ($0\leq t\leq1$). Its Poincare (period) map, $P\left(R_{0},a\right)$ has its largest eigenvalue , called a Floquet multiplier (see e.g. [12-14]). Function  measures a degree of instability at infection-free equilibrium, separating stable infection-free equilibrium region () from unstable (). Fig. S6(A) shows isocontours of function , with stable infection-free equilibrium region marked in blue (Floquet multiplier <1).

A different procedure is used for stable periodic equilibria $w\left(t,R_{0},a\right)$, where we compute numerically its seasonal mean $\bar{w}\left(R_{0},a\right)$on a suitable grid. The isocontours of $\bar{w}\left(R_{0},a\right)$ are shown in Fig.S6 (B). Mathematically, one would expect two regions, () and ($\bar{w}\left(R_{0},a\right)>0$) to complement each other. But numerically a discrepancy arises between approximate isocontours , and $\bar{w}\left(R_{0},a\right)\approx0$ - a cusp region of Fig S6 (C). Within the cusp region a gradual transition takes place between unstable infection-free equilibrium (on the left), and positive (but diminishingly small) periodic $w\left(t,R_{0},a\right)$ on the right. Such ‘small periodic equilibria’, while formally stable, have no practical significance. On the one hand, the relevant MWB-values fall below physical limits (e.g. $w<10^{-10}$/host). The corresponding MacDonald system initialized at positive values would slowly relax toward its theoretical ‘periodic equilibrium’, but it would extremely long time to reach it (Fig S6 (D)).

The discrepancy between two stability isocontours explain apparent inconsistency between Figure 1 and Figure 2 in the main paper, where e.g. function $\bar{w}\left(1.5,a\right)$ drops to ‘zero’ before a stable infection-free equilibrium isocontour is reached.

### Periodic mass drug administration in stationary environment for simple MacDonald system

A mass drug administration (MDA) session at time *T* is represented in our setup as instantaneous reduction of mean burden (due to short drug life-time), $w\left(T\right)\to(1-\varepsilon_{H}) w\left(T\right)$, where constant $\varepsilon_{H}$ combines drug efficacy $\varepsilon$ (fraction of killed worms), and the human population coverage fraction $0<f<1$, i.e., $\varepsilon_{H}=\varepsilon f$. Such MDA implies each treatment session reaches a random fraction of the targeted host population. That is, each seasonal MDA can have different treated and untreated populations in the same region.

One can think of such MDA- event as a sharp spike of worm mortality over a short duration, formally represented by a Dirac delta-function.

Each MDA-event is followed by rebound - relaxation towards the endemic state (an equilibrium or a stable periodic cycle). Such relaxation patterns differ between the complete (2D) vs. the reduced MacDonald systems (Fig. S2). A regularly spaced sequence of MDA-events creates yet another type of periodic variability in such dynamic systems. This time periodicity is affected by worm mortality rather than snail population growth/decay. So $\gamma\left(t\right)$ becomes a periodic function with sharp (Dirac delta) spikes, or their finite approximation. As above, one can ask whether such periodic mortality $\gamma\left(t\right)$ can be effectively approximated by its mean value

$$\bar{\gamma}=\gamma\left(1+\frac{1}{T\gamma}\ln\left(1/\varepsilon_{H}\right)\right)$$

Here, the natural $R_{0}$ is replaced by effective reduced value due to ,

$$R_{ef}=\frac{R_{0}}{\left(1+\ln\left(1/\varepsilon_{H}\right)/T\gamma\right)}$$

In particular, we ask whether reducing $R_{ef}<1$ (via suitable combination of MDA frequency and efficacy) could lead to elimination. The condition for elimination in the stationary ‘mean’ system is

While the numerator is limited by drug efficacy ($\varepsilon_{H}\leq\varepsilon$) even at 100% coverage, its denominator could be made arbitrarily small by implementation of by sufficiently frequent MDA sessions (small T).

To test the validity of MDA averaging, we ran numeric simulations of two models, periodic MDA, and its ‘average’ model with enhanced worm mortality . The results (Fig. S8) show elimination outcomes for the mean-MDA system, predicted by $R_{ef}<1$, whereas the exact MDA-response curve is locked in a periodic (limit) cycle pattern, even above critical frequency ($T<T_{c}$). Such limit-cycle response patterns arise in many transmission models, including stratified worm burden (SWB) system [10, 11], and they suggest that target reduction goals cannot be met with repeated MDA regimens [15].

### MacDonald system with worm mating: parameter space analysis.

Unlike the simple MacDonald system with its unstable-stable pair of equilibria (‘infection-free and endemic’), the MacDonald system with worm mating [7] is bistable, for sufficiently high$R_{0}>R_{c}$. Specifically, it has ‘infection-free’ and ‘endemic’ states (both stable), plus intermediate breakpoint (saddle) (Fig. S9). For detailed explanation, see ([6, 7]). Another important feature of the MacDonald system with worm mating, shared by stratified worm burden (SWB) system, is its dependence on two dimensionless parameters, $\left(A/\gamma,B/\nu\right)$ , rather than a single $R_{0}$ - their product.

The bistable nature (breakpoint) of MacDonald system with worm mating has implications for MDA control. One such implication concerns regions in the $\left(A/\gamma,B/\nu\right)$ – parameter space that separate the bistable ‘endemic’ state from the ‘infection-free infection’ state. These regions deviate from isocontours of $R_{0}$, so to predict elimination one needs two parameters $\left(A/\gamma,B/\nu\right)$, rather than single $R_{0}$ [7].

Even more stark departures arise for dynamic MDA simulations using models that include mating terms. Here we can use a stationary transmission environment, but vary transmission coefficients. The results (Fig. S10) show large diversity of outcomes, depending on transmission coefficients $\left(A,B\right)$, proportional to population densities ($A\propto N$- snail, $B\propto H$- human). Different ‘snail per human’ ratios ($A/B$) correspond to different transmission environments (population densities), and under identical $R_{0}$ they can produce different outcomes (Fig. S10). In some cases, with a lower snail/human ratio, transmission goes to elimination after a finite number of MDA cycles (though total duration could vary). In other cases, with a higher human/snail ratio, the system is locked in a limit cycle, due to post-MDA rebound.

With such models that included mating factors, we also observed a discrepancy between predictions of the reduced and complete MacDonald systems. Typically, the reduced model would under-predict the MDA response, and show stronger rebound (Fig. S10).

The key conclusions are (i) the conventional $R_{0}$has little predictive value for endemic equilibrium analysis of MacDonald system with worm mating (infection-free-to-endemic transition); (ii) the reduced model (via snail quasi-equilibria) can grossly underestimate MDA responses; (iii) different choices of dimensionless (A,B) transmission coefficients that make up $R_{0}$correspond to different snail-to- human abundance. High *A* (or ratio $N/H$) will produce a stronger post-MDA rebound, and complicate the time and effort required for elimination.

### Analysis of Stratified Worm Burden system

Compared to the simple Macdonald system, an important feature of the stratified worm burden (SWB) system is its dependence on two dimensionless parameters, $\left(A/\gamma,B/\nu\right)$ , rather than their product $R_{0}$. Different ‘snail per human’ population ratios ($A/B$) correspond to different transmission environments. But different choices of (*A,B*) under identical $R_{0}$, can produce different outcomes (Fig. S11). Higher *A* (proportional to snail population density) will make infection more sustainable for both types of seasonality, but the effect is more pronounced for type-II. When the transmission coefficients (*A,B* ) move closer, function $\overline{w}(a)$ becomes less sensitive to seasonal amplitude $a$.

### MDA with systematic non-compliance.

Systematic non-compliance means a fixed host pool remains untreated over the entire MDA program. To simulate such a system we extended the basic MacDonald model, by splitting host population into treated and untreated groups with fractions$\left(f,1-f\right)$, and assigning each one by its MWB variable ,$v(t)$- treated, and$u(t)$- untreated. Both group are identical in term of their exposure and transmission rates. The resulting system of equations is given by

$$\begin{matrix} \frac{dv}{dt}=\gamma\left(y-v\right) \\ \frac{du}{dt}=\gamma\left(y-u\right) \\ \frac{dy}{dt}=\nu\left[R_{0}(fv+\left(1-f\right)u)(1-y)-y\right] \end{matrix}$$

In the absence of MDA, the two groups maintain the same worm burden,$v\left(t\right)=u\left(t\right)$, The differences arise after MDA implementation: after each MDA round, variable $v\left(t\right)$ is reduced to a fraction,$v\left(T\right)\to(1-\varepsilon) v\left(T\right)$ ($\varepsilon$ - drug efficacy), while *u* remains unchanged. The mean worm burden of the entire population, $w=f v+\left(1-f\right) u$, changes accordingly.

We simulated systematic noncompliance system , and compared it to random noncompliance under the same MDA regimen, drug efficacy and coverage level. The key finding was a significant discrepancy between two modes of MDA delivery. The systematic noncompliance can significantly slow the MDA progress. Both groups (*u* and *v)* benefit from MDA, via reduced transmission, though $v\left(t\right)$ drops much faster. But the community MWB, $w\left(t\right)$ lags far behind the comparable ‘random noncompliant’ population (see Fig. S18 and Table S1).

We conducted a more systematic comparison between two modes of delivery, including seasonal MDA timing’ (Table S1).The optimal timing (highlighted column of Table S1) is identical for both strategies close to $\tau\approx1/2$ (mid-season) at large amplitude ($a$),getting shifted toward $\tau\approx3/4$ at moderate values ($a$). Overall, systematic noncompliance lags behind random mode after a 6-year program (Table S1).

The model was implemented and simulated on Wolfram Mathematica platform. Computer codes can be obtained by request (qxh119@case.edu).


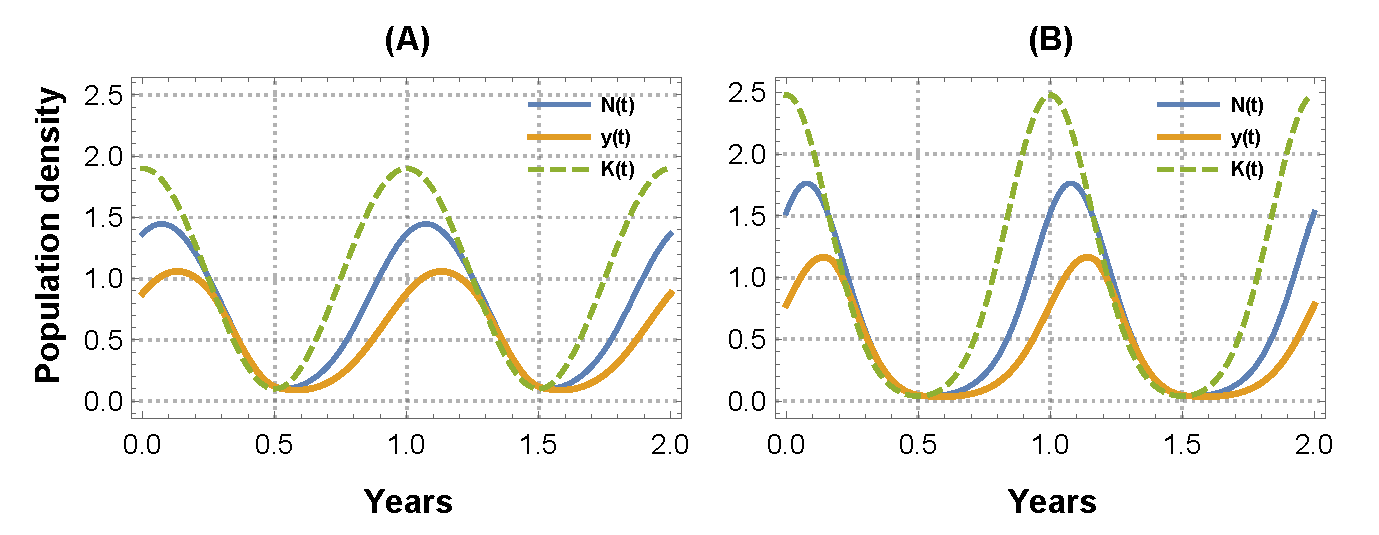


Fig. S1. Typical dynamic seasonal patterns over two-year period for logistic snail model with carrying capacity-function $K\left(t,a\right)$for type I (panel (A)) and type II (panel (B)) model seasonality. Results are shown with properly adjusted snail mortality, i.e.,$y\left(t\right)<N\left(t,a\right)$ for all t, and fixed relative growth rate $\beta/\nu=5$and snail mortality $\nu=4$ /year. The left panel show shows the seasonal MacDonald-type model solution $\left\{N\left(t,a\right),y\left(t\right)\right\}$ for a Type I trigonometric $K\left(t,a\right)$ of amplitude $a=0.9$, and $R_{0}=6$(high transmission intensity). The right panel shows the same for a type II peak-type $K\left(t,a\right)$model with $a=0.6; R_{0}=6$. According to our calculation, seasonal mean values $\bar{N}\left(a\right)$ are reduced compared to the prescribed snail population $N(t,a)$ -case: $\bar{N}=0.73$ (type I, left panel), and $\bar{N}=0.67$(type II, right panel).


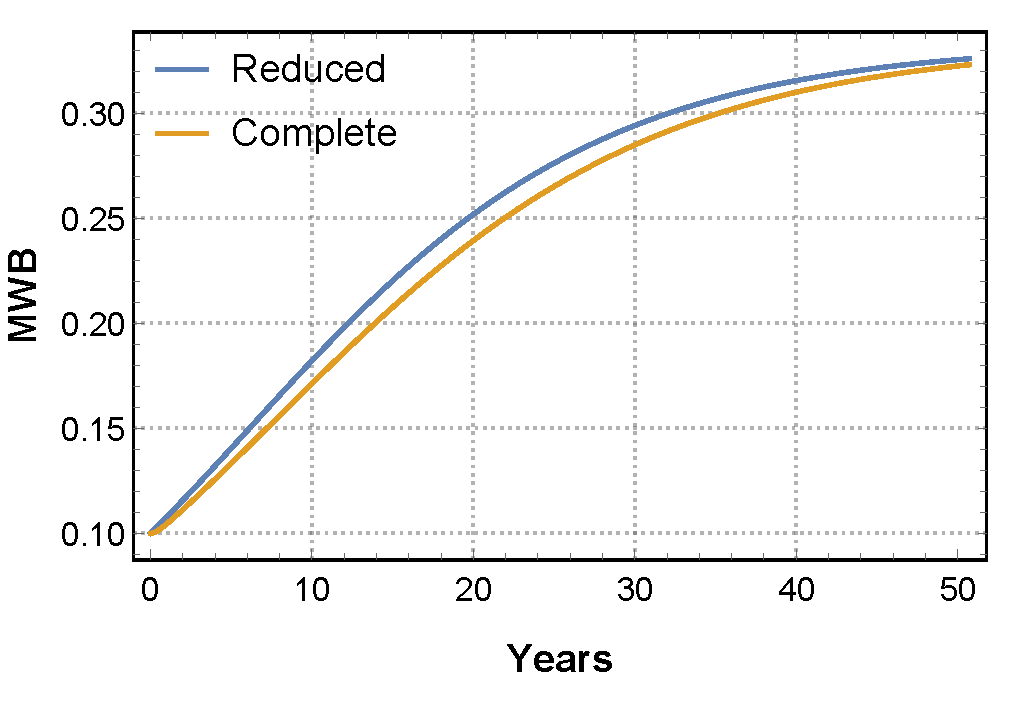


Fig. S2. Comparison of relaxation patterns for stationary MacDonald systems between the reduced one dimensional MacDonald model and the complete two dimensional model. The blue line represents the reduced model and the yellow line represents complete model. Both systems relax to the same equilibrium $w^{*}=1-1/R_{0}$ , but the reduced MacDonald model predicts a faster intermediate relaxation rate. Not shown, these curves also depend on snail mortality $v$, which in this case was $v=2$.

Fig. S3. Periodic solutions over one-year period for mean worm burden $w^{*}\left(t,a\right)$for several choices of $R_{0}$ and amplitude $a$ with a Type I model (trigonometric $N(t,a)$). The left panels are for a reduced (1D) Macdonald-type model system and the right panels are for a complete (2D) MacDonald system, for values of $R_{0}=\{1.5,2,3\}$ and amplitude range $a=\{0,0.2,0.4,0.6,0.95\}$ (blue, yellow, green, red, purple), descending from stationary equilibrium $a=0$ (blue) through $a=0.95$ (purple). Variation of $w^{*}\left(t,a\right)$ about its mean $\bar{w}\left(a\right)=\left\langle w^{*}\left(t,a\right)\right\rangle$ is about 5%-10% for $\left(R_{0},a\right)$, but seasonal average of MWB,$\bar{w}\left(a\right)$, drops below its stationary equilibrium value $w^{*}=1-1/R_{0}$, for all $a>0$.


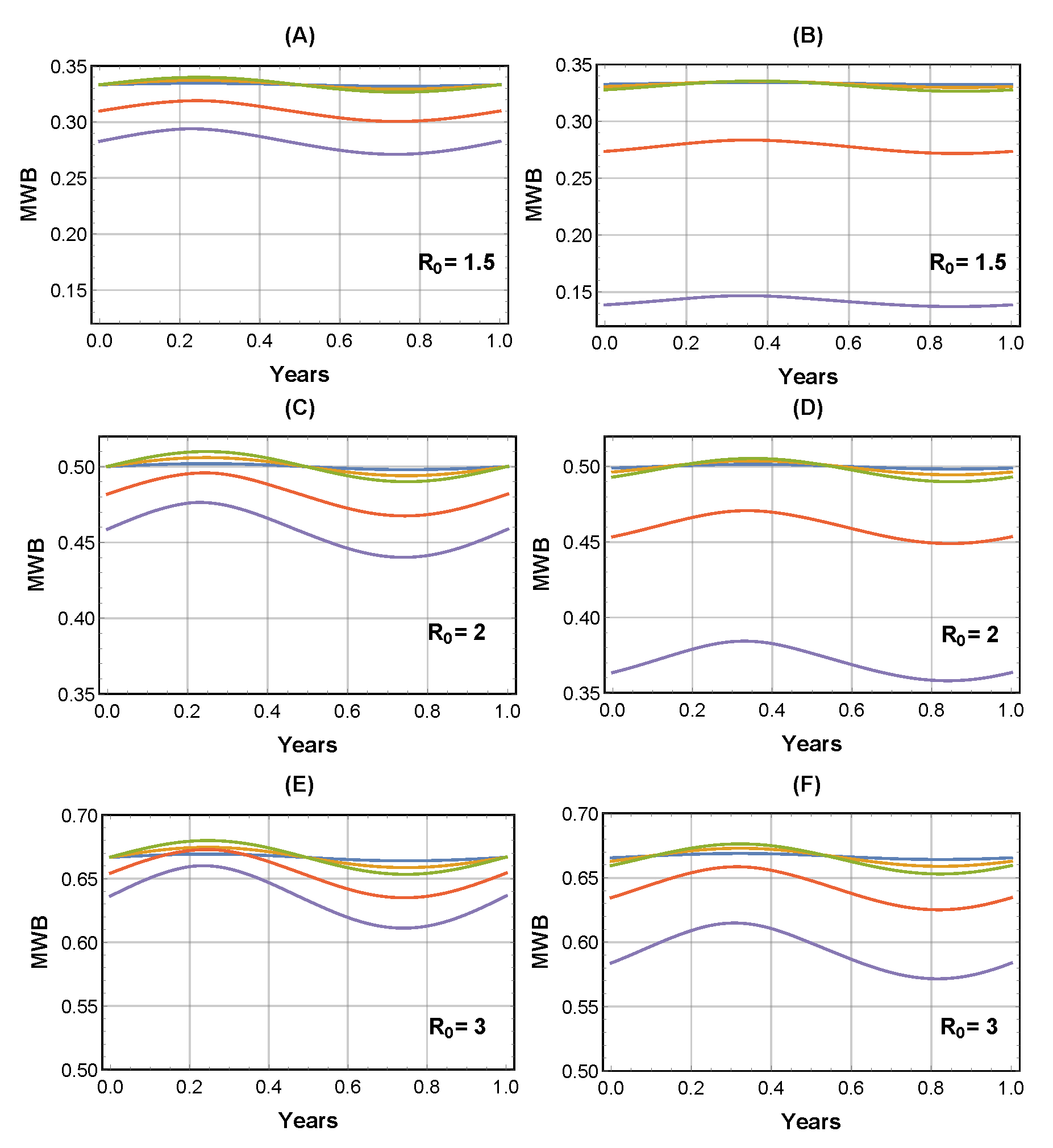


Fig. S4. Periodic solutions for mean worm burden$w^{*}\left(t,a\right)$ for several choices of $R_{0}$ and of amplitude, $a$, using a Type II model with peak $N\left(t,a\right)$. The left panels indicate values for a reduced (1D) Macdonald-type model system, the right panels are for a complete (2D) MacDonald system, for values of $R_{0}=(2,3)$ and amplitude range $a=\{0,0.2,0.4,0.6,0.8\}$ (blue, yellow, green, red, purple).


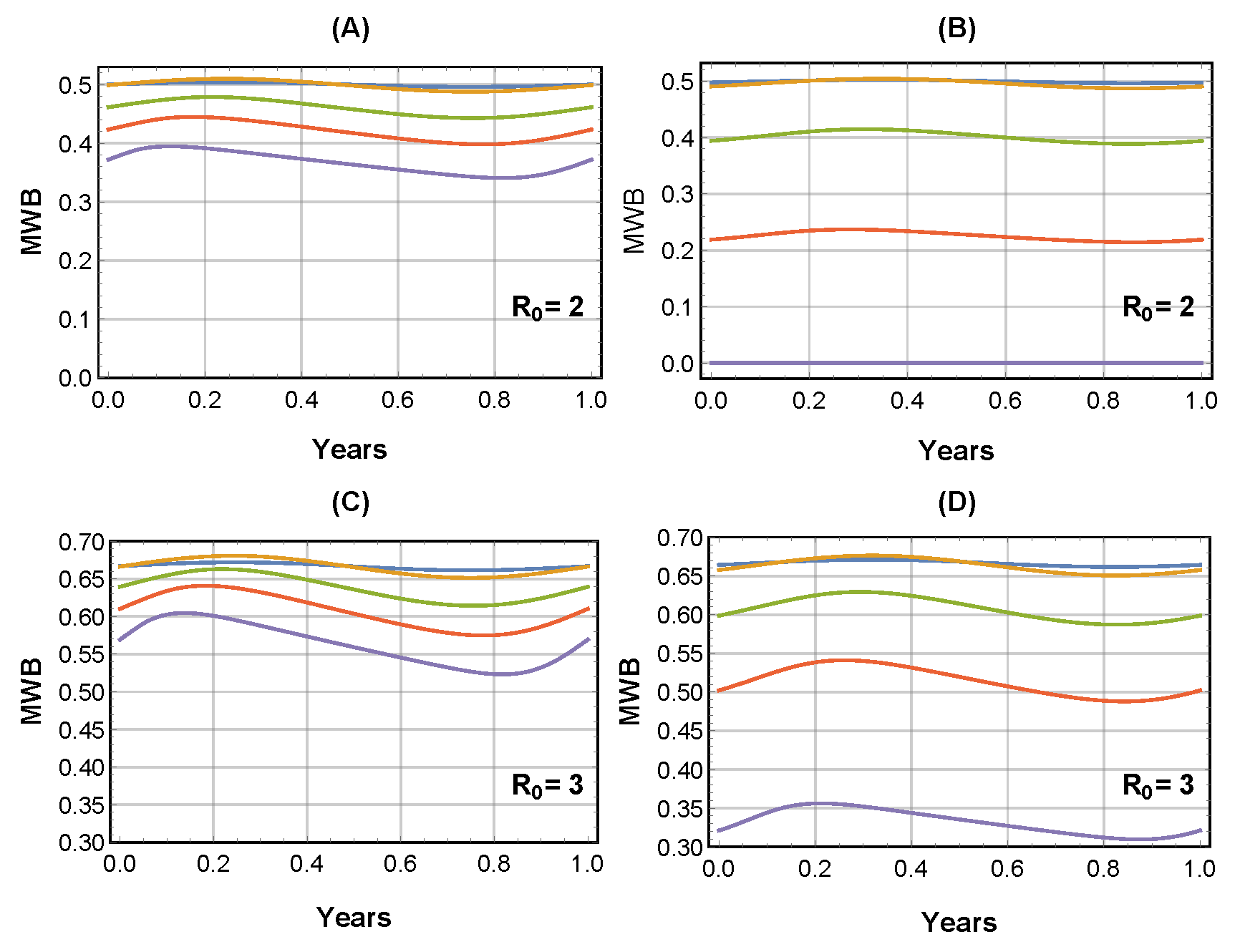

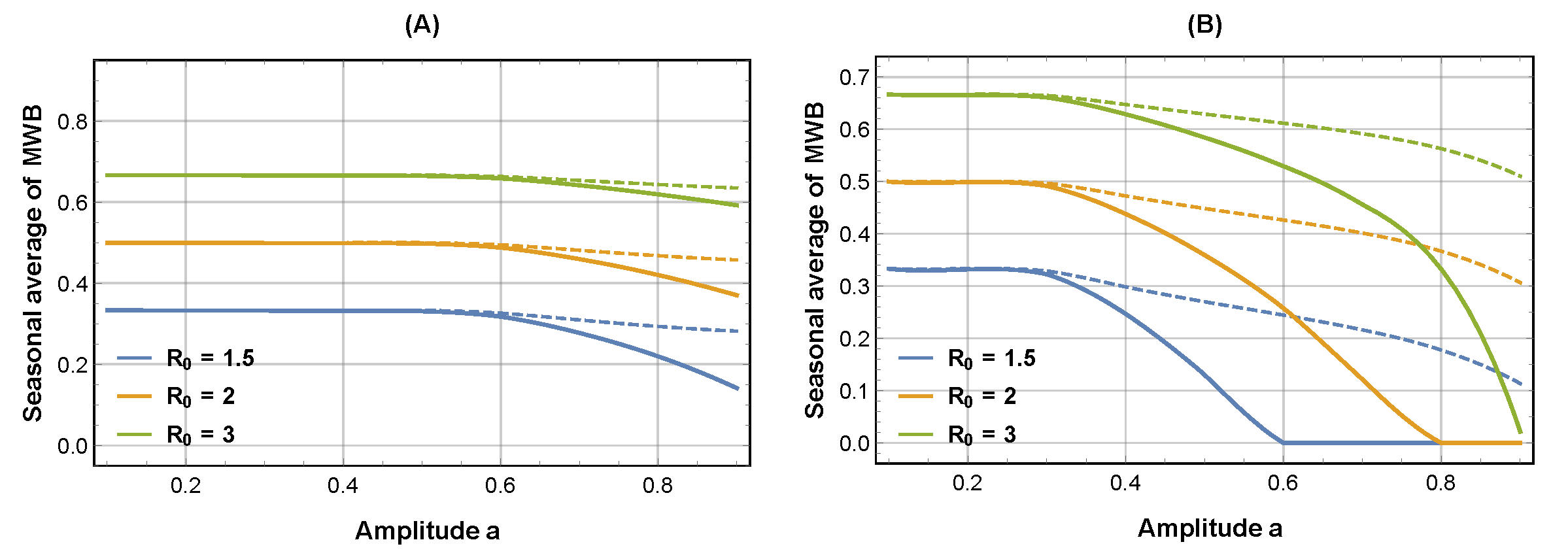


Fig. S5: Seasonal average of MWB, $\bar{w}\left(a\right)=\left\langle w^{*}\left(t,a\right)\right\rangle$ as a function of amplitude $a$ Macdonald system model for type I (panel (A)), and a type II peak (panel (B)). The complete 2D human-snail MacDonald functions (solid) were compared to reduced system (dashed) for 3 values $R_{0}=1.5;2;3$. The Reduced model departed significantly from the full system at larger seasonal amplitudes ($a>0.6$). It can thus over-predict seasonal mean burden by a wide margin.


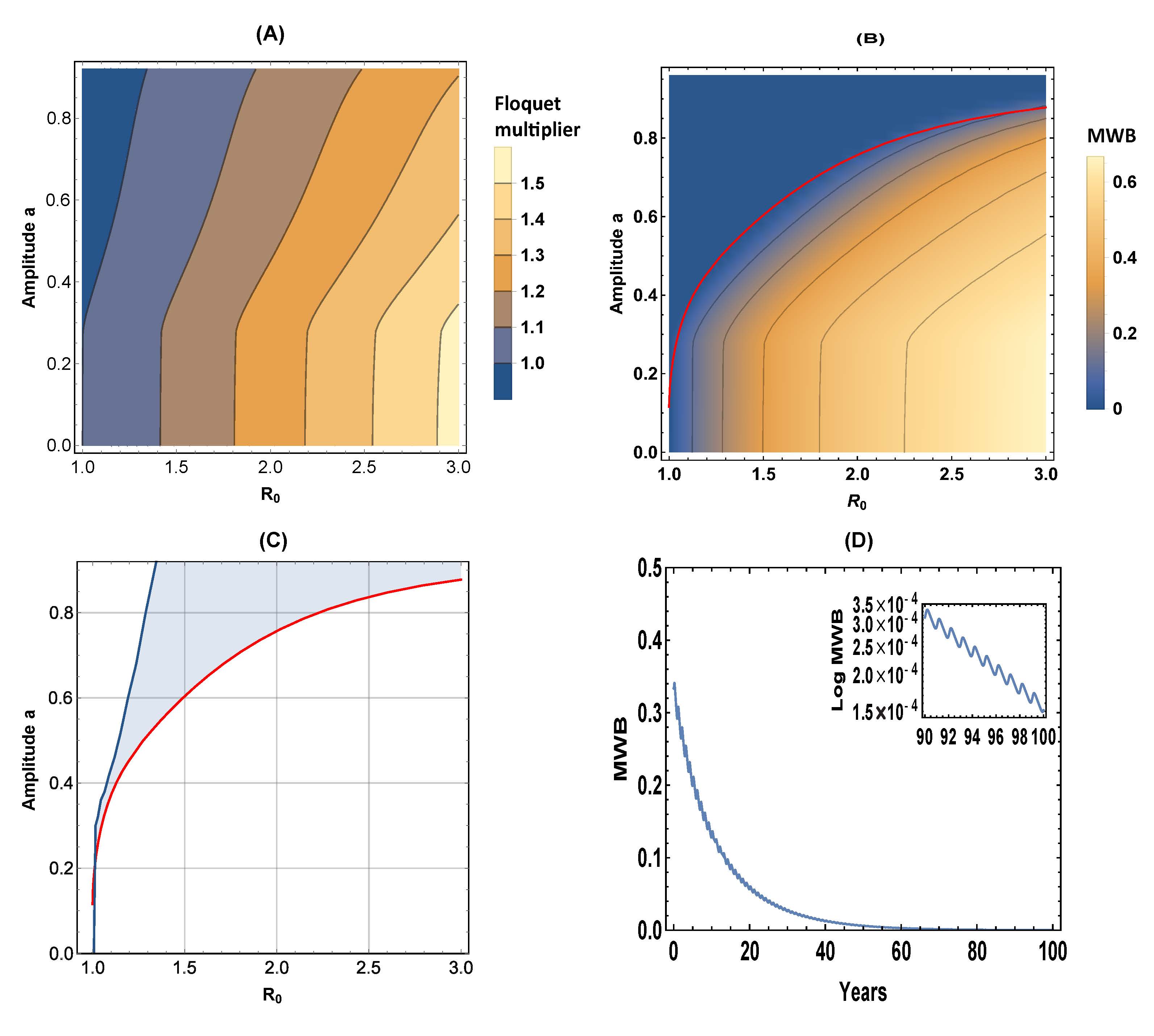


Fig. S6. Parameter space analysis of MacDonald model with type II seasonality. Panel (a) shows isocontours of the Floquet multiple function , that measures the degree of stability of the infection-free (zero) equilibrium, with stable region () marked in dark blue. $\{\left(1,1.05\right);\left(1.05,1.10\right);\left(1.10,1.15\right);\left(1.15,1.20\right);(1.20,\infty)\}$) (light blue-light yellow colors regions) show stable endemic state in which infection persists. Panel B shows a different procedure used for stable periodic equilibria $w\left(t,R_{0},a\right)$ by numerically computing its seasonal mean $\bar{w}\left(R_{0},a\right)$on a suitable grid. The isocontours of $\bar{w}\left(R_{0},a\right)$ are shown. Panel (C) shows a cusp region where numerically a discrepancy arises between approximate isocontours , and $\bar{w}\left(R_{0},a\right)\approx0$. Panel D shows that the corresponding MacDonald system initialized at positive values would slowly relax toward its theoretical ‘periodic equilibrium’, but it would take an extremely long time to reach it. See discussion in Appendix B for details.


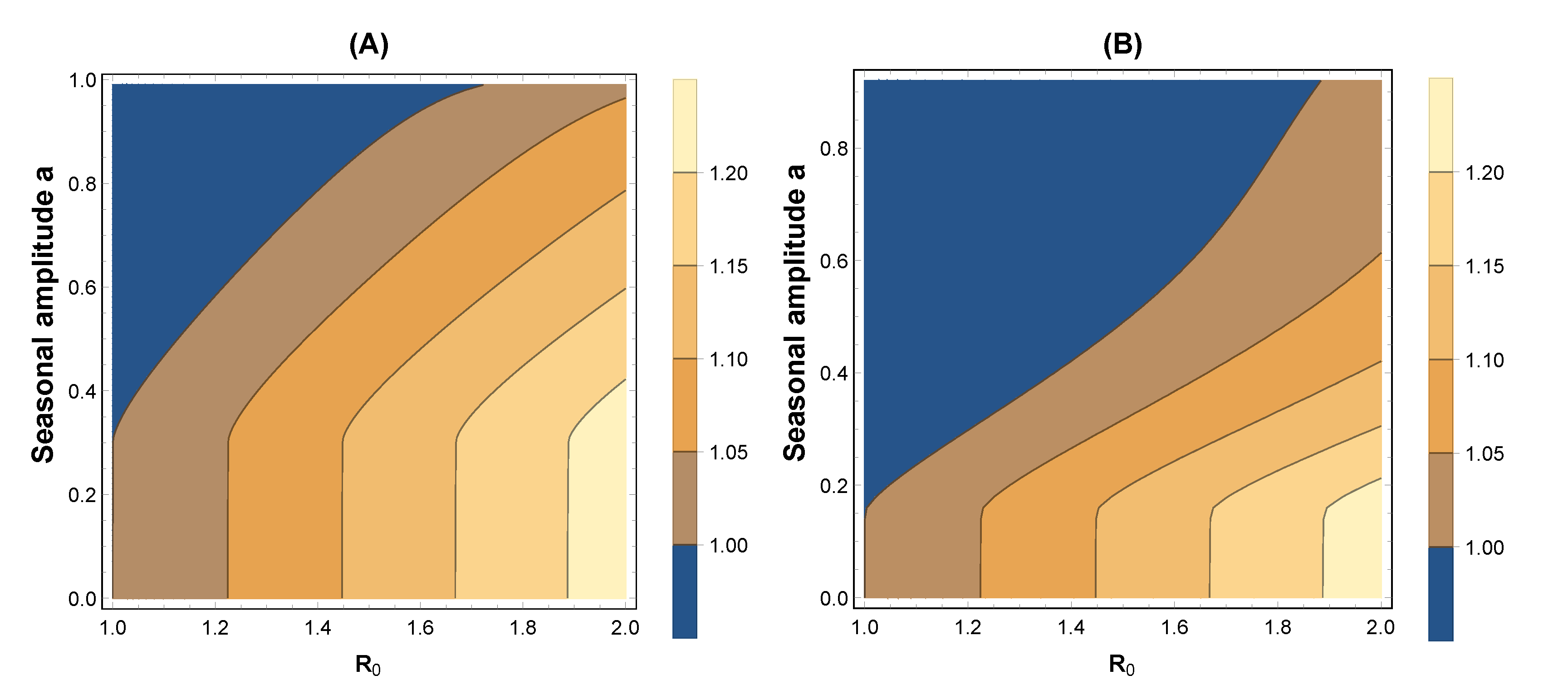


Fig. S7: Isocontours of the Floquet multiple function , that measure the degree of stability of the infection-free (zero) equilibrium, with stable region () marked in dark blue. $\{\left(1,1.05\right);\left(1.05,1.10\right);\left(1.10,1.15\right);\left(1.15,1.20\right);(1.20,\infty)\}$) (light blue-light yellow colors regions) show stable endemic state in which infection persist. Not shown, these curves also depend on snail mortality, which in this case was $v=2$*(see $v=4$) in main paper*.


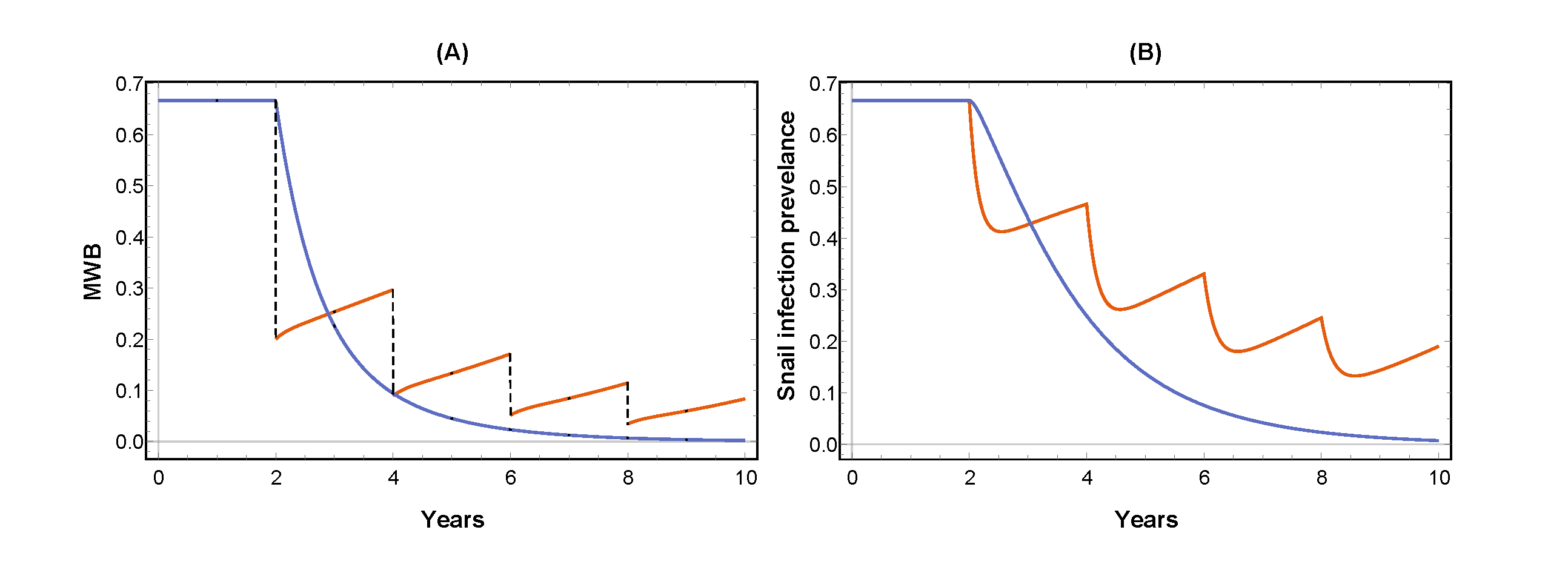


Fig. S8. Comparison of the predictions of human and snail infection prevalence during periodic MDA using the simple MacDonald-type model. The figures show the effects of averaging to indicate MDA impact: each panel compares the predicted impact of repeated periodic MDA using a periodic step-wise model (blue), vs. the effects estimated by a corresponding ‘mean drug clearing’ model (yellow). The former approaches a limit cycle regime of persisting prevalence, the latter goes to elimination of infection (effective BRN $R_{ef}<1$). The chosen treatment period for these projections is $T=1.7<T_{c}=1.89$.

Fig. S9. Important features of the MacDonald-type model system with mating factor. The left panel shows the (A, B) parameter space of the MacDonald system with mating for rescaled parameters $A\to A/\left(\gamma+\mu\right)$,$B\to B/\nu$. Here Coefficient$A$, is proportional to the snail density coefficient, coefficient $B$depends on the human population size, Coefficients $\gamma$ and $\nu$ are the natural mortality rates for adult worms and snails. Such a MacDonald system with mating that assumes a negative binomial (NB) worm burden distribution requires two dimensionless parameters ($\frac{A}{\gamma},\frac{B}{\nu}$), instead of a single $R_{0}$. In the left panel, the shaded region is ‘stable infection-free’ without endemic equilibrium, whereas the open region has triple (bistable) equilibria. Orange lines are isocontours for $R_{0}=A B=\left\{2.4,2.6,2.8\right\}$. The right panel shows the existence of breakpoints by illustrating functions $F\left(w|A,B\right)$ of the reduced MacDonald system for fixed $R_{0}=2.6$ and three values $A=\{1.5,2,2.5\}$ (blue, yellow, green). Case $R_{0}=2.6; A=1.5$ is fold bifurcation, i.e., transition from stable infection-free to triple (bistable) equilibria).


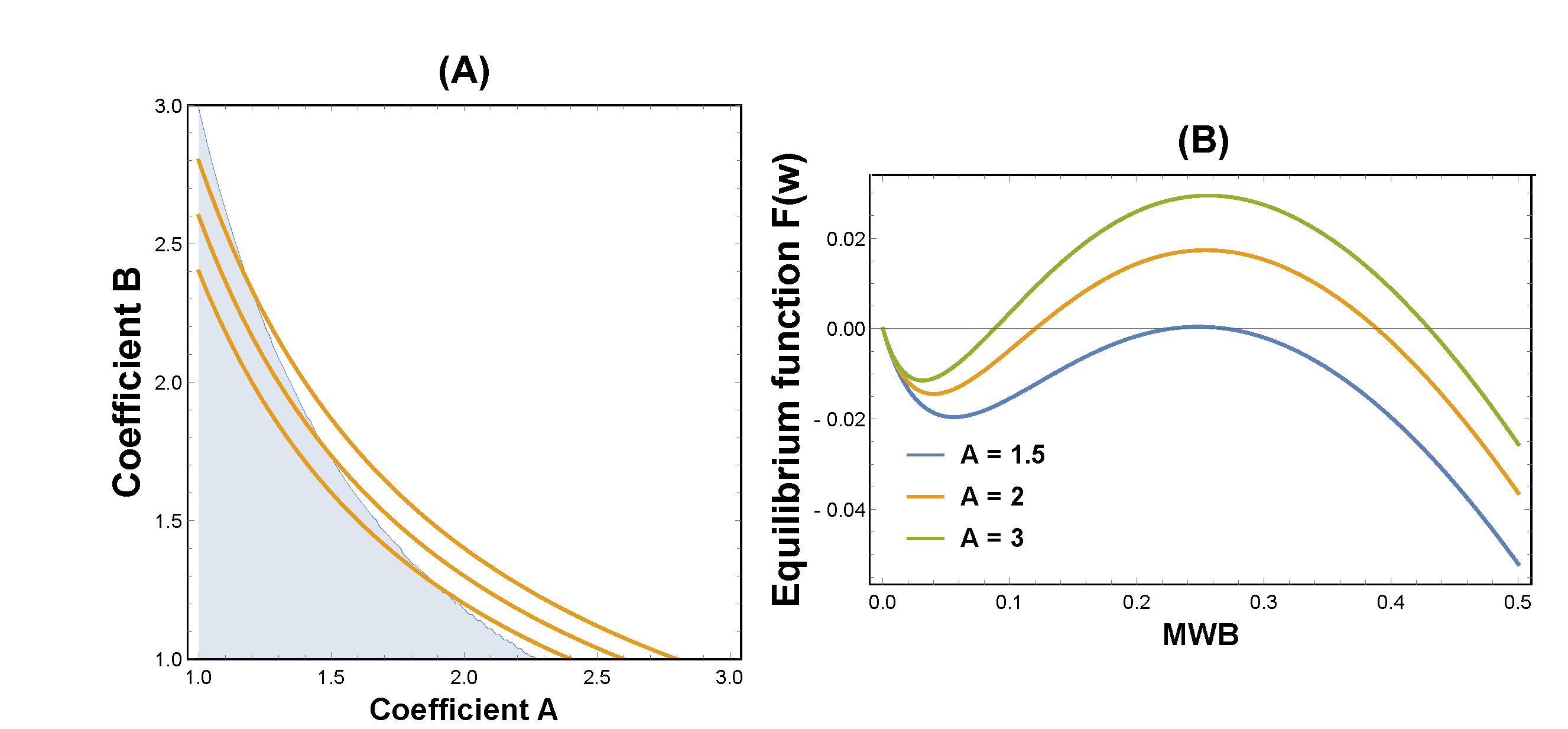

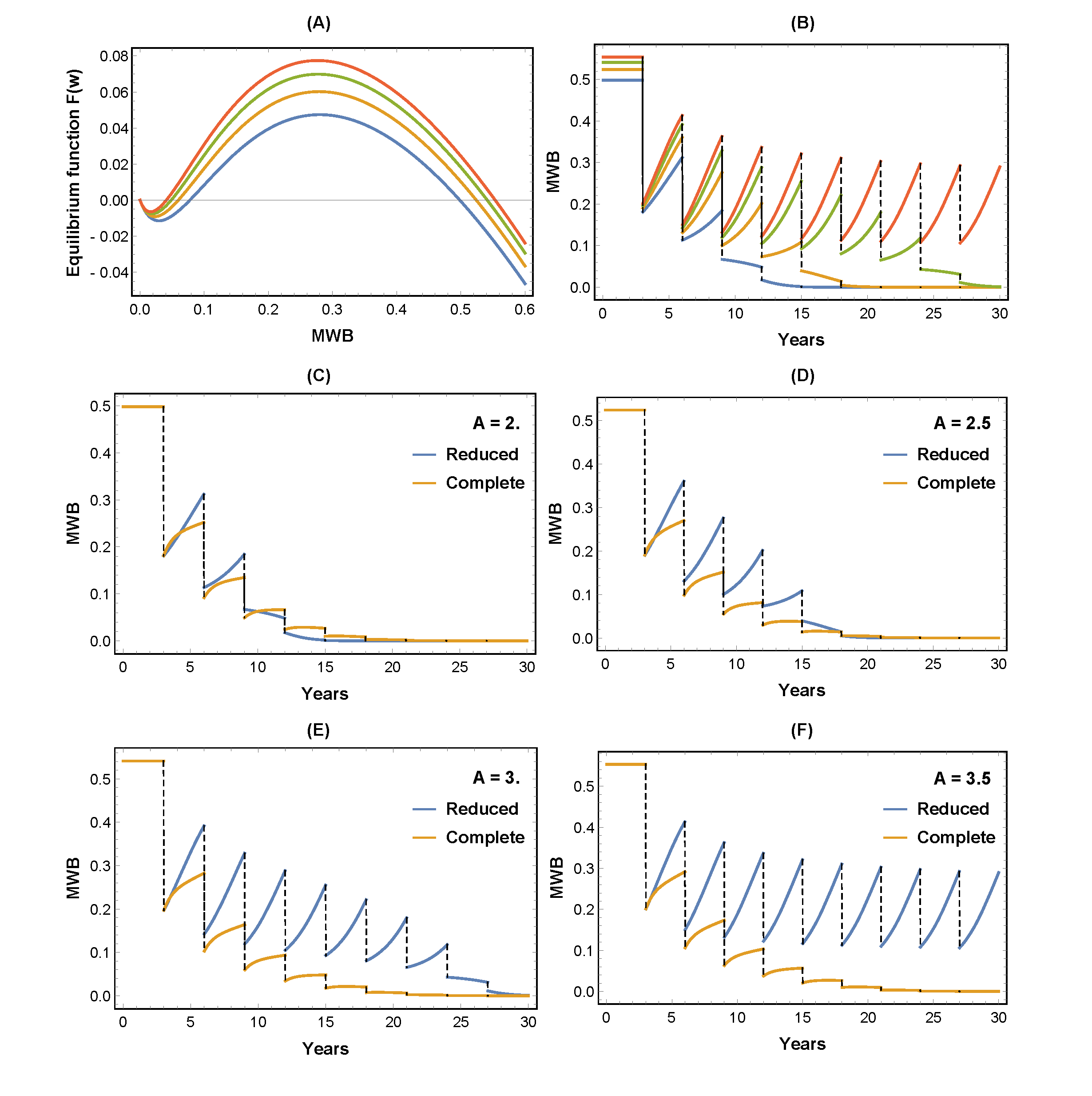


Fig. S10. Periodic MDA control simulated for a stationary MacDonald-type system with mating and negative binomial worm distribution. Panel (a) shows the corresponding reduced MacDonald function F(w), and triple equilibria (at infection-free – at its breakpoint – and endemic). As transmission coefficients are proportional to population densities ($A\propto N$ - snail, $B\propto H$ - human), different (snail per human) A/B ratios correspond to different transmission environments, and under identical $R_{0}$ they can produce different outcomes. We fixed $R_{0}=3$ and chose 4 values of transmission coefficient $A=\left\{2,2.5,3,3.5\right\}$(blue, yellow, green, red). Panel (b) shows MDA histories of 4 communities as predicted by reduced MacDonald systems. In all four scenarios, disease is eliminated in finite time once prevalence falls below the predicted breakpoint. However, a higher A (from a large snail-to-human ratio) manifests stronger rebound and requires more MDA rounds to achieve elimination. Panels (c-f) compare MDA histories of the reduced standard MacDonald model (blue) and the complete model (yellow), for 4 choices of transmission coefficient$A=\{2,2.5,3,3.5\}$, respectively. The two models have comparable predictions at low transmission coefficient, $A=2$ (panel (c)), but they diverge markedly as A increases. In most cases the reduced model exhibits stronger post-MDA rebound, and therefore takes longer to eliminate transmission, i.e., to reach a breakpoint. The extreme case is $A=3.5$ (panel (f)), where the complete model goes to elimination after 25 years of MDA, whereas the reduced model is locked in a permanent limit cycle of community worm burden.

Fig. S11. Seasonal average of MWB, $\bar{w}\left(a\right)=\left\langle w^{*}\left(t,a\right)\right\rangle$ as a function of amplitude $a$ for a stratified worm burden (SWB) system. Left panel shows type I trigonometric $N\left(t,a\right)$case, and right panel a type II peak $N(t,a)$. The transmission intensity (dimensionless parameter) $R_{o}$ is proportional to the product of $\left(A,B\right)$. Coefficient$A$, is proportional to the snail density coefficient, *N*, coefficient $B$depends on the human population size *H*. Under identical $R_{0}$, we examined three different choices of $(A,B)$ values, which correspond to different transmission environments (population densities). Case 1,2, and 3 corresponds to $\{A=1,B=2\}$, $\{A=\frac{3}{2},B=\frac{4}{3}\}$, and $\{A=2,B=1\}$, respectively. Higher *A* (or snail density) will have more sustainable infection for both type I and type II seasonality, and even more pronounced for type-II seasonality.


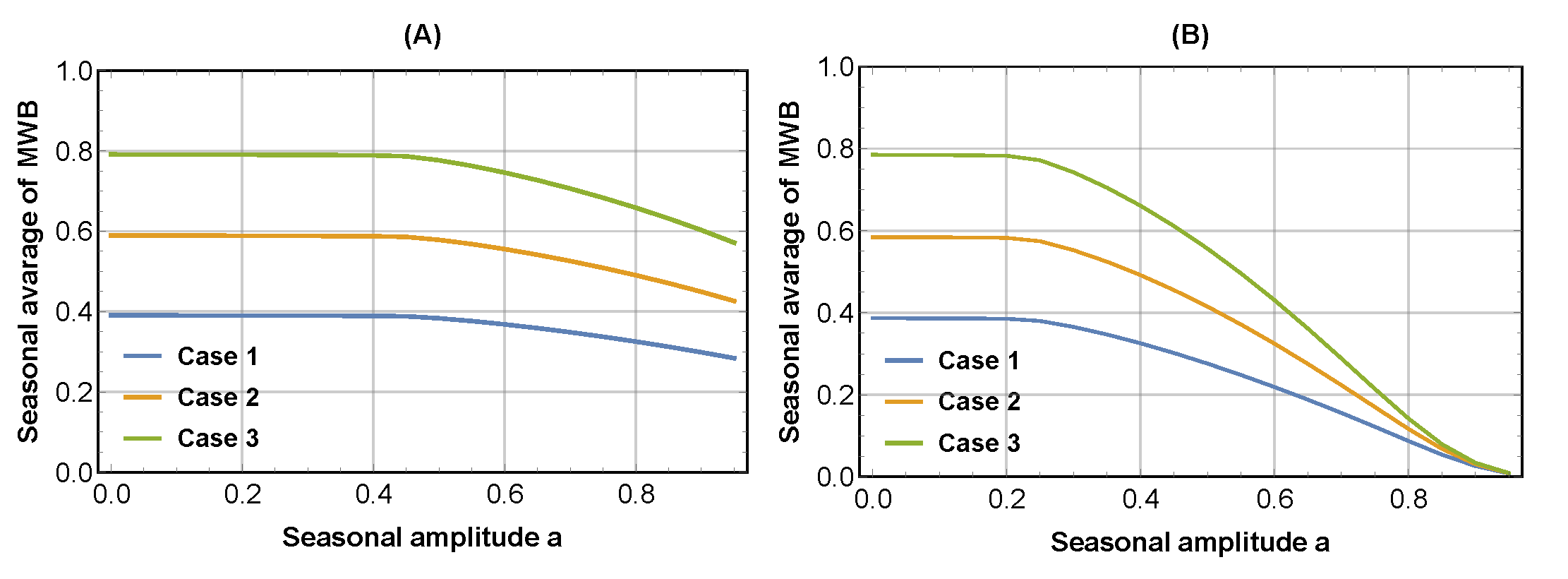

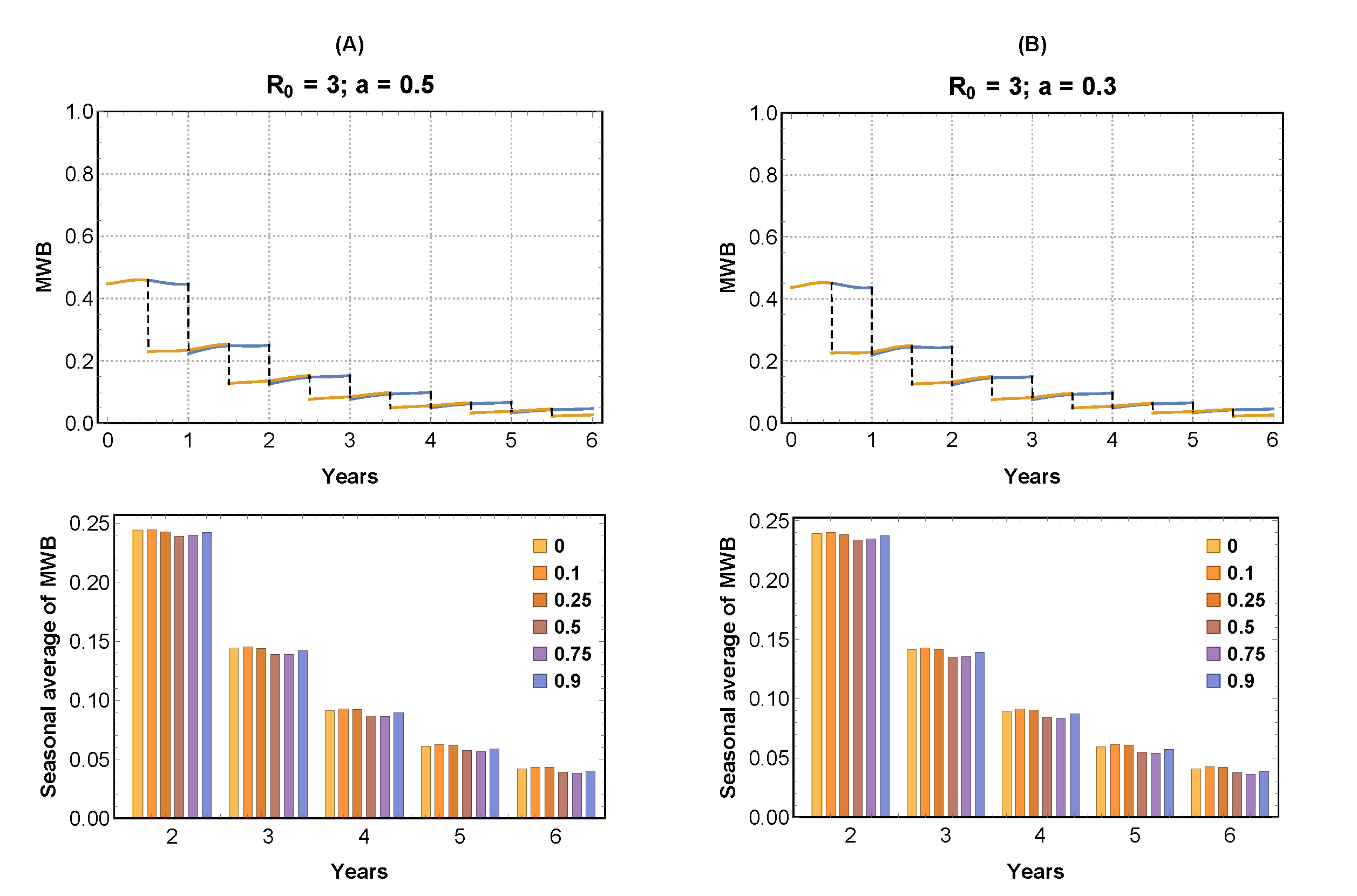


Fig. S12. Effect of annual (random non-compliance) MDA on seasonal MWB patterns for two types of seasonality at moderate transmission environment $R_{0}=3$ and moderate seasonal variability. A six year control program was run for MacDonald-type systems having dynamic snail populations (i.e., seasonal CC-functions $K\left(t,a\right)$ of trigonometric type I (left column panel) or peak type II (right column)). Here we used the MDA efficacy $\varepsilon_{H}=50\%$ corresponding to drug efficacy ($\varepsilon=0.75-0.85$, i.e., 70-85% reduction in MWB) and the MDA treatment population coverage fraction ($f\approx0.6-0.7$). The upper panels show six-year histories with MDA given at the start of each season $\tau=0$ (blue), or at mid-season $\tau=1/2$(yellow) . The lower panels show the five-year seasonal averages for MWB among local humans when different seasonal timings ($0\leq\tau<1$ ) were used for implementation. For each seasonal timing,$\tau=\{0,\frac{1}{10},\frac{1}{4},\frac{1}{2},\frac{3}{4},\frac{9}{10}\}$, we averaged MWB over the proper one year time interval, i.e., over the period $\left(\tau,\tau+1\right)$, for successive six years.

Fig. S13. Effect of molluscicide timing on transmission dynamics of MacDonald-type systems with seasonal snail populations having carrying capacity $K\left(t,a\right)$of trigonometric type I (left column), or peak type II (right column) at moderate transmission environment $R_{0}=3$ and moderate seasonal variability. Here we used molluscicide efficacy (percent of killing snails) $\varepsilon_{S}=70\%.$ Two colors correspond to different seasonal timing of molluscicide application: at the start of the season, $\tau=0$ (blue), or at mid-season, $\tau=1/2$ (yellow). The effect of seasonal timing on long-term patterns of transmission (6-year history) can be significant: $\tau=0$ implementation ultimately gives higher worm-burden reduction among local humans than does $\tau=1/2$ implementation.


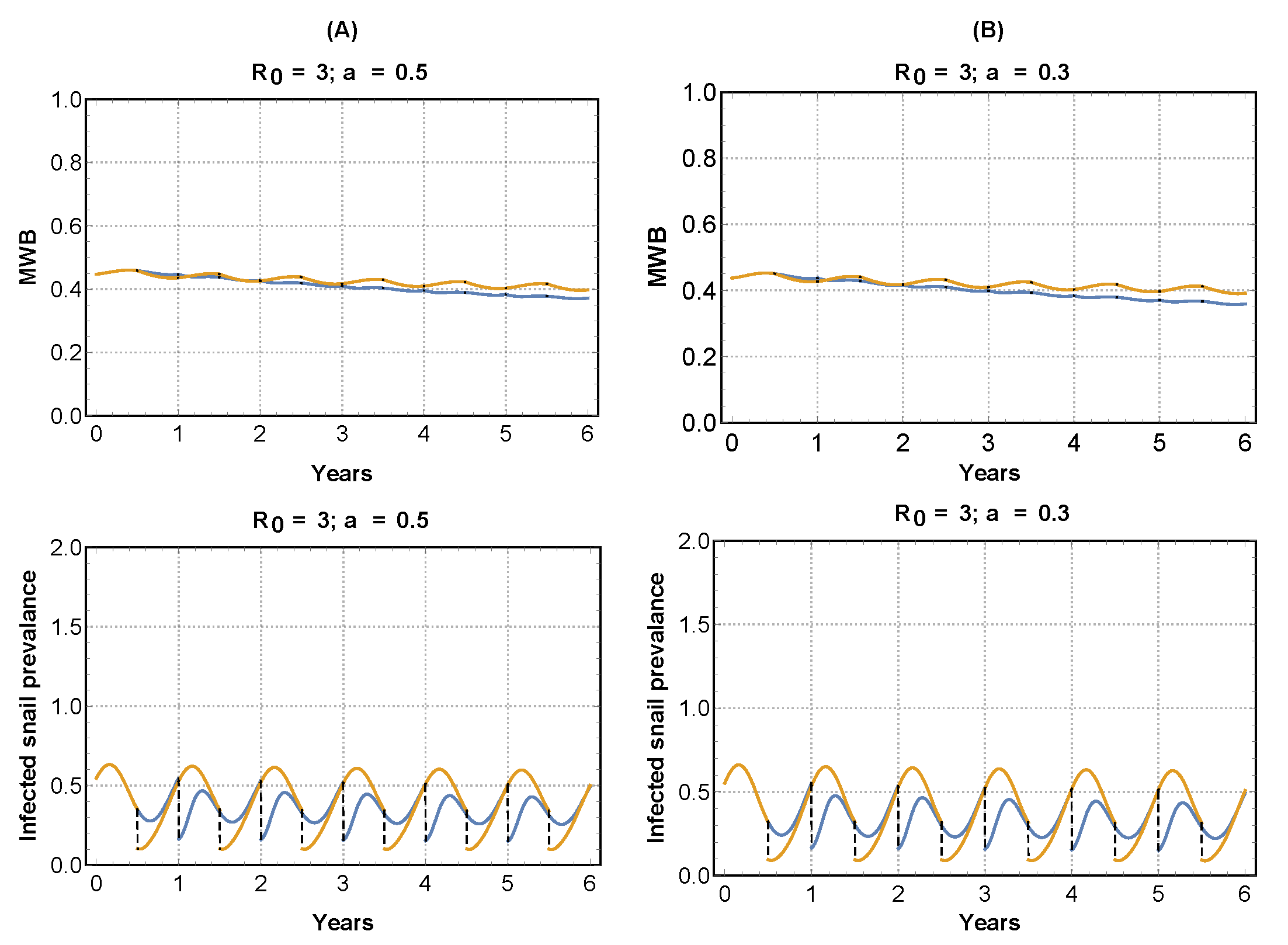


Fig. S14. Effect of annual (random non-compliance) MDA on seasonal MWB patterns for two types of seasonality at extreme high transmission environment $R_{0}=6$ and seasonal variability. A six-year control program was run for MacDonald-type systems having dynamic snail populations as mentioned in Fig. S12. Here we used the MDA efficacy $\varepsilon_{H}=50\%$. The upper panels show six-year histories with MDA given at the start of each season $\tau=0$ (blue), or at mid-season $\tau=1/2$(yellow). The lower panels show seasonal average MWB for different MDA timing $\tau=\{0,\frac{1}{10},\frac{1}{4},\frac{1}{2},\frac{3}{4},\frac{9}{10}\}$. Qualitatively, these results look similar to $R_{0}=3$ case discussed in the paper, but the seasonal difference is more pronounced.


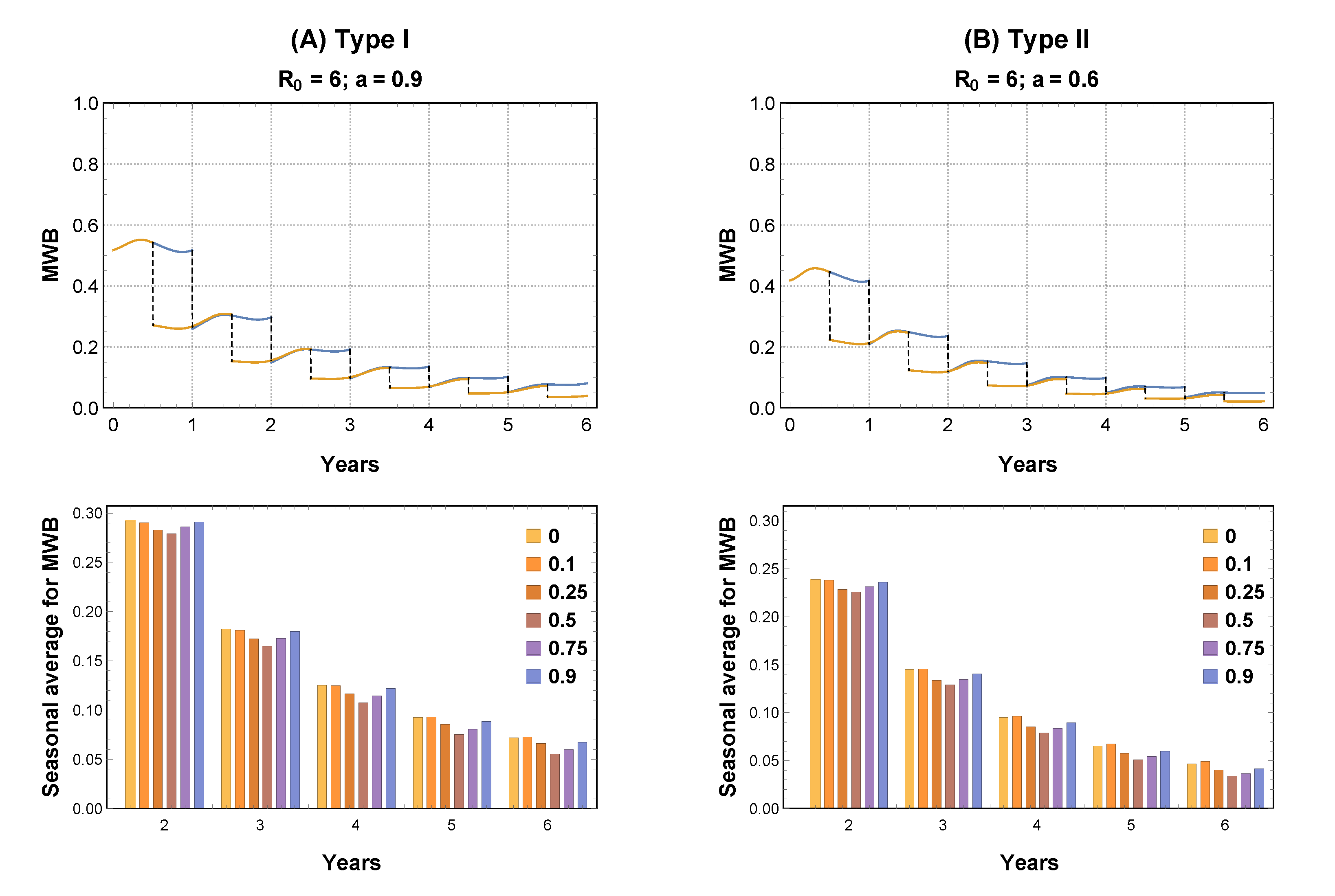

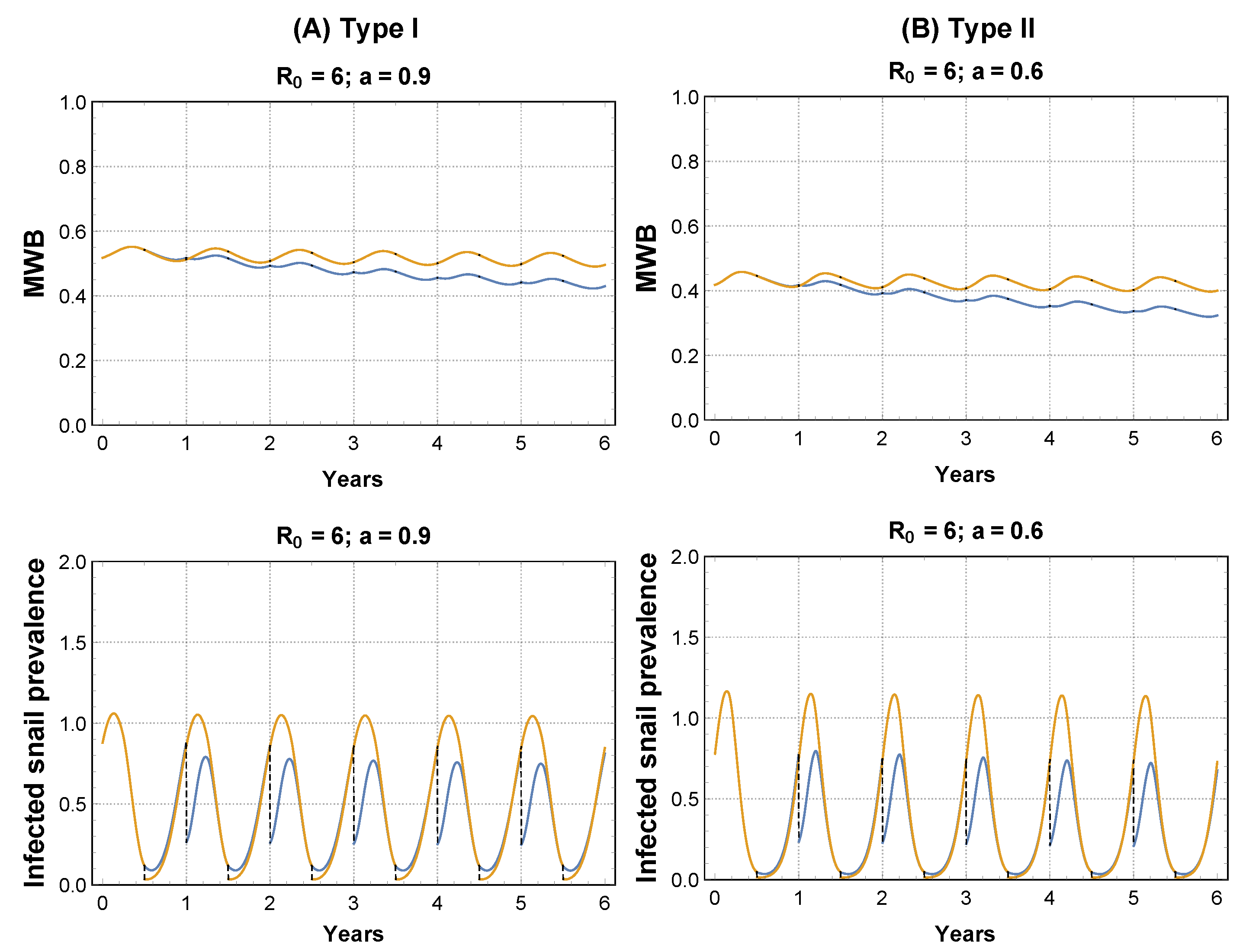


Fig. S15 Effect of molluscicide timing on transmission dynamics of MacDonald-type systems for high intensity transmission ($R_{0}=6$), with seasonal snail populations having carrying capacity $K\left(t,a\right)$of type I (left column), or type II (right column). Here we used molluscicide efficacy $\varepsilon_{S}=70\%.$ Two colors correspond to different seasonal timing of molluscicide application: at the start of the season, $\tau=0$ (blue), or at mid-season, $\tau=1/2$ (yellow). The effect of seasonal timing on long-term patterns of transmission (6-year history) can be significant: $\tau=0$ implementation ultimately gives higher worm-burden reduction among local humans than does $\tau=1/2$ implementation. Qualitatively, these results look similar to $R_{0}=3$ case discussed in the paper, but the seasonal difference is more significant.

**
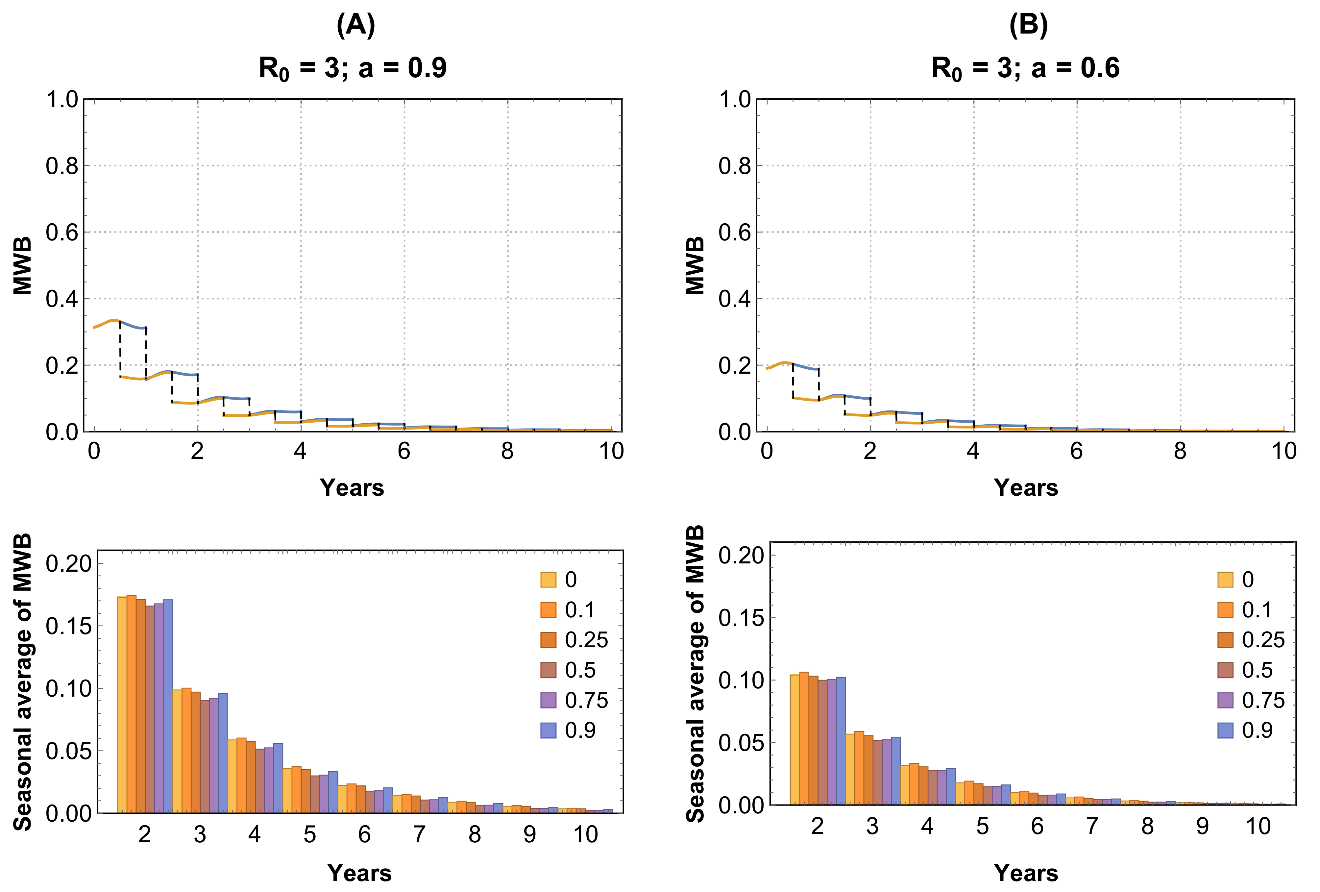
**

Fig. S16. Effect of annual (random non-compliance) MDA on seasonal MWB patterns for two types of seasonality at moderate transmission environment $R_{0}=3$ and high seasonal variability. A ten-year control program was run for MacDonald-type systems having dynamic snail populations (i.e., seasonal CC-functions $K\left(t,a\right)$ of trigonometric type I (left column panel) or peak type II (right column)). Here we used the MDA efficacy $\varepsilon_{H}=50\%$. The upper panels show six-year histories with MDA given at the start of each season $\tau=0$ (blue), or at mid-season $\tau=1/2$ (yellow) . The lower panels show seasonal average MWB for different MDA timing $\tau=\{0,\frac{1}{10},\frac{1}{4},\frac{1}{2},\frac{3}{4},\frac{9}{10}\}$.


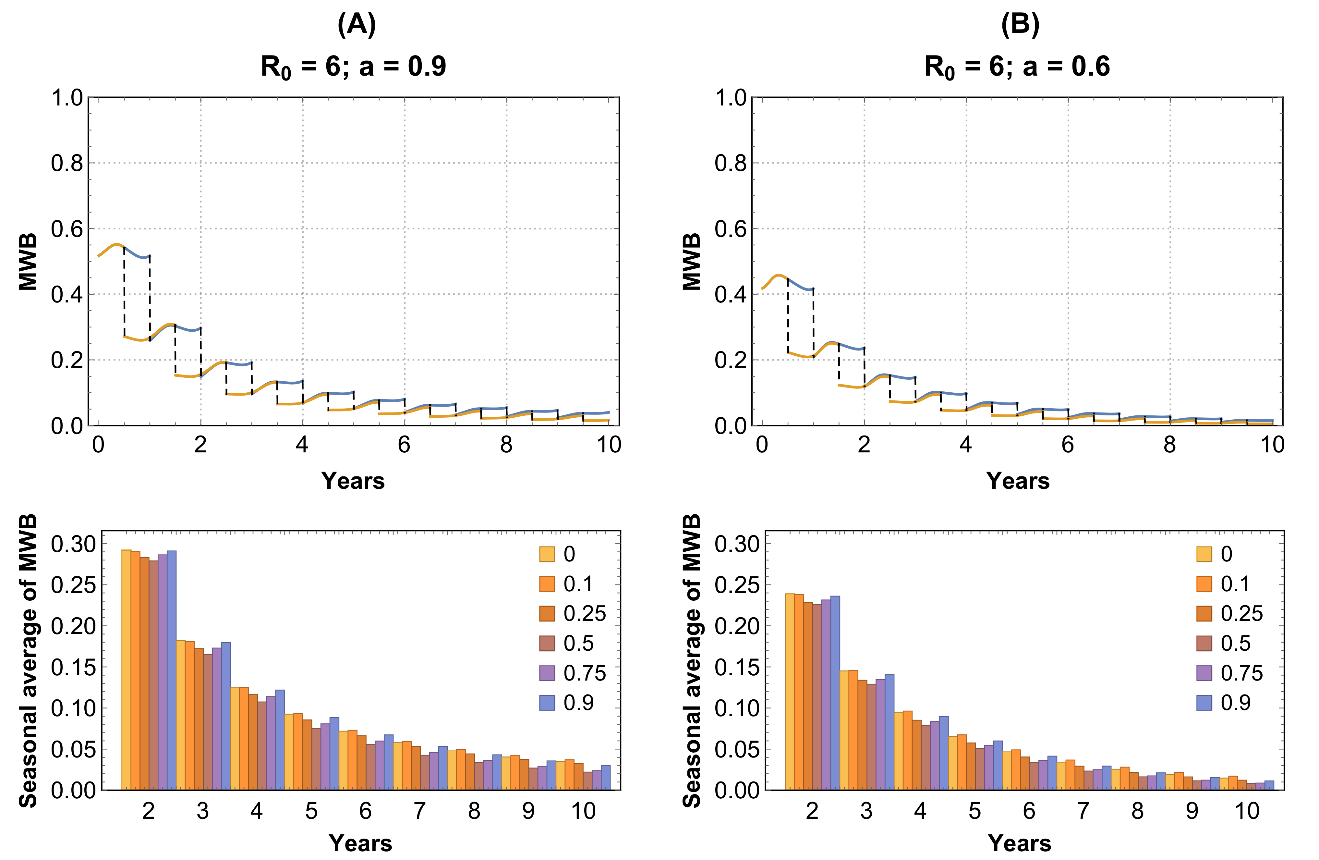


Fig. S17. Annual MDA with random non-compliance, on seasonal MWB patterns for high transmission intensity ($R_{0}=6$), and high seasonal amplitude. A ten-year control program was run for MacDonald-type systems with logistic snail population (carrying capacity function $K\left(t,a\right)$ of type I (left column panel) or type II (right column)). We used MDA efficacy parameter $\varepsilon_{H}=50\%$. The upper panels show typical histories initiated at start of season $\tau=0$ (blue), and mid-season $\tau=1/2$ (yellow). The lower panels show seasonal average MWB for different MDA timing $\tau=\{0,\frac{1}{10},\frac{1}{4},\frac{1}{2},\frac{3}{4},\frac{9}{10}\}$ .


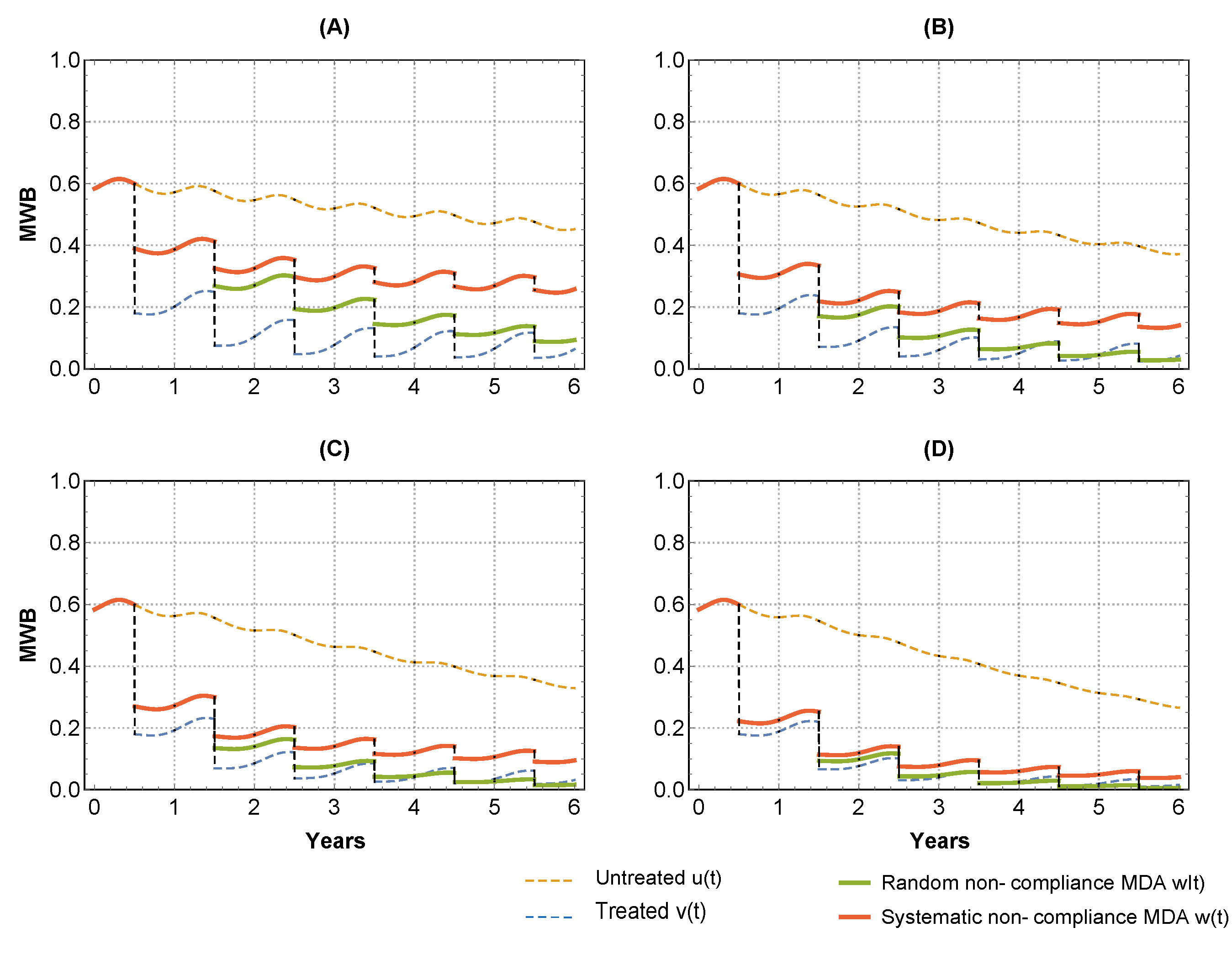


Fig. S18: Typical trajectories of annual MDA program for two modes of drug delivery: systematic and random non-compliance. For comparison, we fixed model inputs and parameters: (transmission intensity $R_{0}=3$, seasonal amplitude $a=0.9$, several choices of MDA efficacy $\varepsilon_{H}=\{35\%,50\%,55\%,63\%\}$, and population coverage fraction $f=\{0.5;0.7;0.786;0.9\}$ (Panel A-Panel D).

The blue and yellow curves are MWB-functions of the treated group ($v\left(t\right)$ and untreated group $u\left(t\right)$ in the systematic non-compliant mode. The red curve is the corresponding weighted mean of treated and untreated groups with the systematic non-compliance MDA, and the green curve is the result with random non-compliance MDA. The results indicate that, at the typical level of program compliance (< 90%), the effect of systematic non-compliance MDA is overall worse than rotating (random) non-compliance MDA.

Table S1: Comparison between random and systematic non-compliance for a 6-year annual MDA program. We assessed the efficacy of the program via mean worm burden (MWB) percent reduction ‘year 6’/’year1’, $\bar{w}\left(Y_{6},\tau\right)/\bar{w}\left(Y_{0},\tau\right)$. Both modes of MDA delivery, the basic MacDonald system and its extension , were simulated under similar conditions: $R_{0}=3$, drug efficacy $\varepsilon=0.7$, and population coverage fraction $f=0.7$. We simulated intra-seasonal timing values, $\tau=\{0,\frac{1}{10},\frac{1}{4},\frac{1}{2},\frac{3}{4},\frac{9}{10}\}$. The optimal choice (highlighted) was consistent for two MDA delivery modes. It was close to mid-season $\tau\approx1/2$ for strong seasonality (amplitude $a$) and shifted toward $\tau\approx3/4$ at moderate seasonal amplitude ($a$). Overall, systematic non-compliance lags behind in the burden reduction, compared to random (rotating) MDA regimens.

|  | | Time points within the seasonal cycle for MDA $\tau$ | | | | | |
| --- | --- | --- | --- | --- | --- | --- | --- |
|  |  | 0 | 1/10 | 1/4 | 1/2 | 3/4 | 9/10 |
| **Type I**  Strong Seasonality Amplitude $a=0.9$ | Random  non-compliance  MDA | 87.1% | 86.5% | 82.7% | 89.3% | 89.2% | 88.1% |
|  | Systematic  non-compliance  MDA | 69.4% | 69.0% | 69.5% | 71.0% | 70.8% | 70.0% |
| **Type II**  Strong Seasonality Amplitude $a=0.6$ | Random  Non-compliance  MDA | 90.3% | 89.6% | 90.7% | 92.7% | 92.0% | 91.3% |
|  | Systematic  Non-compliance  MDA | 76.2% | 75.9% | 76.6% | 77.4% | 77.3% | 76.8% |

Table S2: Effect of annual random non-compliance MDA in terms of MWB reduction among local human populations for transmission intensity$R_{0}=3$. We simulated a ten-year MDA-regimen for a MacDonald-type model system having dynamic snail populations (seasonal carrying capacity (CC) function $K\left(t,a\right)$ of trigonometric types I or peak type II). Impact was measured in terms of relative MWB reduction for local residents (“Year 10 over endemic Year 0”) using seasonal average values $\bar{w}\left(Y_{10},\tau\right)/\bar{w}\left(Y_{0},\tau\right)$of MWB. We examined several different values of intra-seasonal timing for MDA, $\tau=\{0,\frac{1}{10},\frac{1}{4},\frac{1}{2},\frac{3}{4},\frac{9}{10}\}$,as fractions of the seasonal cycles , three possible levels of MDA efficacy (instantaneous reduction of mean worm burden) $\varepsilon_{H}=\left\{50\%,55\%,70\%\right\}$as combined effect of drug efficacy (fraction of killed worms) and population coverage fraction, and two values of seasonal amplitude, $a$. The optimal timing (highlighted) varied in the range $1/2<\tau<3/4$. It was close to mid-season $\tau\approx1/2$ in the presence of strong seasonal amplitude ($a$) and shifted toward $\tau\approx3/4$ at moderate seasonal amplitude ($a$).

|  | | MDA efficacy$\varepsilon_{H}$ | Time points within the seasonal cycle for MDA $\tau$ | | | | | |
| --- | --- | --- | --- | --- | --- | --- | --- | --- |
|  |  |  | 0 | 1/10 | 1/4 | 1/2 | 3/4 | 9/10 |
| **Type I** | Strong  Seasonality  Amplitude $a=0.9$ | 50% | 97.9% | 97.7% | 97.9% | 98.6% | 98.6% | 98.3% |
|  |  | 55% | 98.9% | 98.8% | 98.9% | 99.3% | 99.3% | 99.1% |
|  |  | 70% | 99.9% | 99.9% | 99.9% | 100.0% | 100.0% | 99.9% |
|  | Moderate  Seasonality  Amplitude $a=0.5$ | 50% | 95.3% | 95.0% | 94.9% | 95.7% | 96.1% | 95.7% |
|  |  | 55% | 97.3% | 97.0% | 96.9% | 97.6% | 97.8% | 97.6% |
|  |  | 70% | 99.7% | 99.6% | 99.6% | 99.7% | 99.8% | 99.7% |
| **Type II** | Strong  Seasonality  Amplitude $a=0.6$ | 50% | 99.0% | 98.8% | 99.0% | 99.3% | 99.3% | 99.2% |
|  |  | 55% | 99.5% | 99.4% | 99.5% | 99.7% | 99.7% | 99.6% |
|  |  | 70% | 100.0% | 99.9% | 100.0% | 100.0% | 100.0% | 100.0% |
|  | Moderate  Seasonality  Amplitude $a=0.3$ | 50% | 95.5% | 95.1% | 95.0% | 95.9% | 96.3% | 95.9% |
|  |  | 55% | 97.4% | 97.1% | 97.0% | 97.7% | 98.0% | 97.7% |
|  |  | 70% | 99.7% | 99.6% | 99.6% | 99.7% | 99.8% | 99.8% |

Table S3: For transmission intensity$R_{0}=3$, effect of annual molluscicide application in terms of mean worm burden (MWB) reduction in the human host population. A ten-year control program was simulated using a MacDonald-type model system having a dynamic snail population seasonality with carrying capacity (CC)-function $K\left(t,a\right)$ of trigonometric type I or peak type II structure. As for Table S2, progress was measured by relative seasonal average reduction of MWB $\bar{w}\left(Y_{10},\tau\right)/\bar{w}\left(Y_{0},\tau\right)$. We examined several possible intra-seasonal timings for molluscicide application,$\tau=\{0,\frac{1}{10},\frac{1}{4},\frac{1}{2},\frac{3}{4},\frac{9}{10}\}$, as fractions of the seasonal cycle, three possible levels of molluscicide efficacy $\varepsilon_{S}=\left\{50\%,70\%,90\%\right\}$, and two choices of the seasonal amplitude parameter (moderate or high). Optimal timing for molluscicide control in all cases was $\tau=0$ (highlighted), i.e., at the start/end of the season, when the snail population or its carrying capacity reached its maximum.

|  | | Molluscicide efficacy$\varepsilon_{S}$ | Time points within the seasonal cycle for molluscicide application $\tau$ | | | | | |
| --- | --- | --- | --- | --- | --- | --- | --- | --- |
|  |  |  | 0 | 1/10 | 1/4 | 1/2 | 3/4 | 9/10 |
| **Type I** | Strong  Seasonality  Amplitude $a=0.9$ | 50% | 22.4% | 22.0% | 13.3% | 5.3% | 14.0% | 19.7% |
|  |  | 70% | 33.3% | 32.0% | 19.9% | 9.6% | 22.3% | 30.1% |
|  |  | 90% | 49.3% | 45.6% | 28.4% | 17.6% | 38.6% | 47.2% |
|  | Moderate  Seasonality  Amplitude $a=0.5$ | 50% | 13.9% | 13.9% | 11.9% | 7.7% | 10.1% | 12.7% |
|  |  | 70% | 21.7% | 21.6% | 18.1% | 11.9% | 16.2% | 20.2% |
|  |  | 90% | 34.7% | 33.5% | 27.2% | 19.6% | 28.1% | 33.3% |
| **Type II** | Strong  Seasonality  Amplitude $a=0.6$ | 50% | 32.7% | 30.8% | 16.0% | 11.5% | 22.8% | 29.5% |
|  |  | 70% | 44.7% | 41.7% | 22.2% | 14.8% | 33.3% | 41.5% |
|  |  | 90% | 60.3% | 54.9% | 30.2% | 24.5% | 52.1% | 59.1% |
|  | Moderate  Seasonality  Amplitude $a=0.3$ | 50% | 14.7% | 14.7% | 12.2% | 7.2% | 10.1% | 13.3% |
|  |  | 70% | 23.0% | 22.8% | 18.4% | 11.1% | 16.4% | 21.2% |
|  |  | 90% | 36.6% | 35.1% | 27.5% | 18.5% | 28.9% | 35.0% |

Table S4: Effect of annual random non-compliance MDA in terms of mean worm burden (MWB) reduction among local human populations for an extreme high transmission intensity$R_{0}=6$. We simulated a six-year MDA-regimen for a MacDonald-type model system having dynamic snail populations. Impact was measured in terms of relative MWB reduction for local residents (“Year 6 over endemic Year 0”) using seasonal average values $\bar{w}\left(Y_{6},\tau\right)/\bar{w}\left(Y_{0},\tau\right)$of MWB. We examined several different values of intra-seasonal timing for MDA, $\tau=\{0,\frac{1}{10},\frac{1}{4},\frac{1}{2},\frac{3}{4},\frac{9}{10}\}$,as fractions of the seasonal cycles, three possible levels of MDA efficacy $\varepsilon_{H}=\left\{50\%,55\%,70\%\right\}$ and two values of seasonal amplitude, $a$. The optimal timing (highlighted) varied in the range $1/2<\tau<3/4$. It was close to mid-season $\tau\approx1/2$ in the presence of strong seasonal amplitude ($a$) and shifted toward $\tau\approx3/4$ at moderate seasonal amplitude ($a$).

|  | | MDA efficacy$\varepsilon_{H}$ | Time points within the seasonal cycle for MDA $\tau$ | | | | | |
| --- | --- | --- | --- | --- | --- | --- | --- | --- |
|  |  |  | 0 | 1/10 | 1/4 | 1/2 | 3/4 | 9/10 |
| **Type I** | Strong  Seasonality  Amplitude $a=0.9$ | 50% | 75.4% | 74.9% | 76.6% | 80.1% | 79.1% | 76.9% |
|  |  | 55% | 79.9% | 79.2% | 80.9% | 84.6% | 83.9% | 81.5% |
|  |  | 70% | 90.8% | 89.8% | 91.0% | 94.6% | 94.4% | 92.5% |
|  | Moderate  Seasonality  Amplitude $a=0.5$ | 50% | 70.5% | 70.1% | 70.5% | 72.6% | 72.6% | 71.3% |
|  |  | 55% | 74.7% | 74.2% | 74.5% | 76.8% | 77.0% | 75.7% |
|  |  | 70% | 85.8% | 84.9% | 84.8% | 87.5% | 88.5% | 87.1% |
| **Type II** | Strong  Seasonality  Amplitude $a=0.6$ | 50% | 80.5% | 79.4% | 82.3% | 84.9% | 84.3% | 82.4% |
|  |  | 55% | 84.8% | 83.7% | 86.4% | 89.1% | 88.6% | 86.8% |
|  |  | 70% | 94.3% | 93.1% | 95.0% | 97.0% | 96.9% | 95.8% |
|  | Moderate  Seasonality  Amplitude $a=0.3$ | 50% | 70.6% | 70.1% | 70.6% | 73.1% | 73.1% | 71.6% |
|  |  | 55% | 74.9% | 74.2% | 74.6% | 77.4% | 77.6% | 76.0% |
|  |  | 70% | 86.0% | 84.9% | 84.7% | 88.0% | 89.1% | 87.5% |

Table S5: Effect of annual molluscicide application in terms of mean worm burden (MWB) reduction in the human host population for high transmission intensity$R_{0}=6$. As for Table S4, a six-year control program was simulated. Progress was measured by relative seasonal average reduction of MWB $\bar{w}\left(Y_{6},\tau\right)/\bar{w}\left(Y_{0},\tau\right)$. We examined several possible intra-seasonal timings for molluscicide application,$\tau=\{0,\frac{1}{10},\frac{1}{4},\frac{1}{2},\frac{3}{4},\frac{9}{10}\}$, as fractions of the seasonal cycle, three possible levels of molluscicide efficacy,$\varepsilon_{S}=\left\{50\%,70\%,90\%\right\}$, and two choices of the seasonal amplitude parameter (moderate or high). Optimal timing for molluscicide control in all cases was $\tau=0$ (highlighted), i.e., at the start/end of the season, when the snail population or its carrying capacity reached its maximum.

|  | | Molluscicide efficacy$\varepsilon_{S}$ | Time points within the seasonal cycle for molluscicide application $\tau$ | | | | | |
| --- | --- | --- | --- | --- | --- | --- | --- | --- |
|  |  |  | 0 | 1/10 | 1/4 | 1/2 | 3/4 | 9/10 |
| **Type I** | Strong  Seasonality  Amplitude $a=0.9$ | 50% | 8.5% | 7.8% | 4.1% | 1.5% | 5.5% | 7.7% |
|  |  | 70% | 13.7% | 12.3% | 6.5% | 2.7% | 9.5% | 12.8% |
|  |  | 90% | 22.9% | 19.6% | 10.0% | 6.4% | 18.3% | 22.6% |
|  | Moderate  Seasonality  Amplitude $a=0.5$ | 50% | 5.9% | 5.6% | 4.2% | 2.6% | 4.4% | 5.6% |
|  |  | 70% | 9.5% | 8.9% | 6.6% | 4.3% | 7.4% | 9.2% |
|  |  | 90% | 16.3% | 14.8% | 10.7% | 7.9% | 13.8% | 16.3% |
| **Type II** | Strong  Seasonality  Amplitude $a=0.6$ | 50% | 11.5% | 9.9% | 3.3% | 1.6% | 7.3% | 10.3% |
|  |  | 70% | 18.2% | 15.4% | 5.4% | 2.9% | 12.7% | 17.0% |
|  |  | 90% | 29.5% | 23.8% | 8.4% | 7.4% | 24.6% | 29.5% |
|  | Moderate  Seasonality  Amplitude $a=0.3$ | 50% | 6.3% | 5.9% | 4.2% | 2.4% | 4.5% | 5.9% |
|  |  | 70% | 10.2% | 9.4% | 6.6% | 3.9% | 7.6% | 9.8% |
|  |  | 90% | 17.4% | 15.5% | 10.6% | 7.3% | 14.4% | 17.3% |

Table S6: For an extreme high transmission intensity$R_{0}=6$, comparison of optimal-control progress for (random) mass drug administration (MDA) alone vs. an integrated MDA + molluscicide-based snail control strategy. As in Tables S4 and S5, its progress was measured by relative seasonal average reduction of MWB $\bar{w}\left(Y_{6},\tau\right)/\bar{w}\left(Y_{0},\tau\right)$. A six-year control program was simulated for a MacDonald-type model system having dynamic snail populations ($K\left(t,a\right)$of type-I or type-II), three levels of MDA efficacy $\varepsilon_{H}=\left\{50\%,55\%,70\%\right\}$, three levels of molluscicide efficacy $\varepsilon_{S}=\left\{50\%,70\%,90\%\right\}$, and two different amplitudes of seasonality. The impact for MDA-only was estimated at its optimal time of delivery, as suggested by Table S4. The impact of the MDA+snail strategy was based on separate delivery at their individual optimal timings, $\tau$, as suggested by Table S1 and Table S, namely $\tau=1/2$ or $3/4$ for MDA, and $\tau=0$ for molluscicide application.

| **Type I** |  | Strong Seasonality Amplitude $a=0.9$ | | | | Moderate Seasonality Amplitude $a=0.5$ | | | |
| --- | --- | --- | --- | --- | --- | --- | --- | --- | --- |
|  | MDA efficacy$\varepsilon_{H}$ | MDA only | MDA+snail control$\varepsilon_{S}=\left\{50\%,70\%,90\%\right\}$ | | | MDA only | MDA+snail control $\varepsilon_{S}=\left\{50\%,70\%,90\%\right\}$ | | |
|  | $50\%$ | 80.1% | 91.1% | 92.8% | 94.9% | 72.6% | 85.7% | 87.7% | 90.6% |
|  | $55\%$ | 84.6% | 93.9% | 95.2% | 96.7% | 77.0% | 89.0% | 90.7% | 93.2% |
|  | $70\%$ | 94.6% | 98.7% | 99.0% | 99.4% | 88.5% | 96.1% | 96.9% | 98.0% |
| **Type II** |  | Strong Seasonality Amplitude $a=0.6$ | | | | Moderate Seasonality Amplitude $a=0.3$ | | | |
|  | MDA efficacy$\varepsilon_{H}$ | MDA only | MDA+snail control$\varepsilon_{S}=\left\{50\%,70\%,90\%\right\}$ | | | MDA only | MDA+snail control $\varepsilon_{S}=\left\{50\%,70\%,90\%\right\}$ | | |
|  | $50\%$ | 84.9% | 93.9% | 95.1% | 96.7% | 73.1% | 86.2% | 88.2% | 91.1% |
|  | $55\%$ | 89.1% | 96.1% | 97.0% | 97.9% | 77.6% | 89.5% | 91.2% | 93.6% |
|  | $70\%$ | 97.0% | 99.3% | 99.5% | 99.7% | 89.1% | 96.3% | 97.2% | 98.2% |

SI References

1. Macdonald G. The dynamics of helminth infections, with special reference to schistosomes. Transactions of the Royal Society of Tropical Medicine and Hygiene **1965**; 59:489-506.

2. Nasell I. A hybrid model of schistosomiasis with snail latency. Theoretical Population Biology **1976**; 10:47-69.

3. May RM. Togetherness among schistosomes: its effects on the dynamics of the infection. Mathematical Biosciences **1977**; 35:301-43.

4. Nasell I. Mating models for schistosomes. Journal of Mathematical Biology **1978**; 6:21-35.

5. Anderson R, May R. Herd immunity to helminth infection and implications for parasite control. Nature **1985**; 315:493-6.

6. Anderson RM, May RM. Infectious Diseases of Humans: Dynamics and Control. Oxford University Press, **1992**.

7. Gurarie D, King CH. Population biology of *Schistosoma* mating, aggregation, and transmission breakpoints: more reliable model analysis for the end-game in communities at risk. PLoS One **2014**; 9:e115875.

8. Mari L, Ciddio M, Casagrandi R, et al. Heterogeneity in schistosomiasis transmission dynamics. Journal of Theoretical Biology **2017**; 432:87-99.

9. Gurarie D, Lo NC, Ndeffo-Mbah ML, Durham DP, King CH. The human-snail transmission environment shapes long term schistosomiasis control outcomes: implications for improving the accuracy of predictive modeling. PLoS Neglected Tropical Ddiseases **2018**; 12:e0006514.

10. Gurarie D, King CH, Wang X. A new approach to modelling schistosomiasis transmission based on stratified worm burden. Parasitology **2010**; 137:1951-65.

11. Gurarie D, King CH, Yoon N, Li E. Refined stratified-worm-burden models that incorporate specific biological features of human and snail hosts provide better estimates of *Schistosoma* diagnosis, transmission, and control. Parasite Vector **2016**; 9.

12. Mari L, Casagrandi R, Bertuzzo E, Rinaldo A, Gatto M. Floquet theory for seasonal environmental forcing of spatially explicit waterborne epidemics. Theoretical Ecology **2014**; 7:351-65.

13. Klausmeier CA. Floquet theory: a useful tool for understanding nonequilibrium dynamics. Theoretical Ecology **2008**; 1:153-61.

14. Bacaër N, Guernaoui S. The epidemic threshold of vector-borne diseases with seasonality. Journal of Mathematical Biology **2006**; 53:421-36.

15. Li EY, Gurarie D, Lo NC, Zhu X, King CH. Improving public health control of schistosomiasis with a modified WHO strategy: a model-based comparison study. The Lancet Global Health **2019**; 7:e1414-e22.
